# The impact of 100% electrification of domestic heat in Great Britain

**DOI:** 10.1016/j.isci.2023.108239

**Published:** 2023-10-18

**Authors:** Vassilis M. Charitopoulos, Mathilde Fajardy, Chi Kong Chyong, David M. Reiner

**Affiliations:** 1 Judge Business School, University of Cambridge, Trumpington Street, Cambridge CB2 1AG, UK; 2 Department of Chemical Engineering, Sargent Centre for Process Systems Engineering, University College London, Torrington Place, London WC1E 7JE, UK; 3 Center on Global Energy Policy, School of International and Public Affairs, Columbia University, 1255 Amsterdam Avenue, New York, NY 10027, USA

**Keywords:** Energy systems, Energy management, Energy Modelling

## Abstract

Britain has been a global leader in reducing emissions, but little progress has been made on heat, which accounts for almost one-third of UK emissions and the largest single share is domestic heat, which is responsible for 17% of the national total. Given the UK’s 2050 “Net-Zero” commitment, decarbonizing heat is becoming urgent and currently one of the main pathways involves its electrification. Here, we present a spatially explicit optimization model that investigates the implications of electrifying domestic heat on the operation of the power sector. Using hourly historical gas demand data, we conclude that the domestic peak heat demand is almost 50% lower than widely cited values. A 100% electrification pathway can be achieved with only a 1.3-fold increase in generation capacity compared to a power-only decarbonization scenario, but only by leveraging the role of thermal energy storage technologies without which a further 40% increase would be needed.

## Highlights

• Regional, hourly data reveals lack of synchronicity in domestic peak heat demand
• Overall peak heat demand derived here is almost 50% lower than widely cited values
• Interaction of electrification and renewable distribution leads to decentralized CCS
• Thermal energy storage technologies reduce required capacity additions by up to 40%

## Graphical abstract

**Figure:**
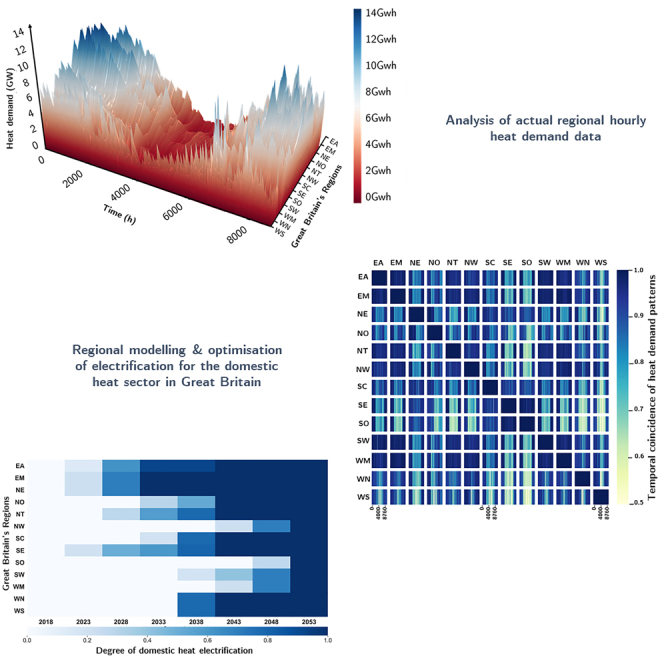

## Introduction

Energy use and emissions from residential heating and cooling are increasingly important in many leading countries in their race to net-zero.^1^^,^^2^ Recent studies on Paris-compliant scenarios indicate considerably less flexibility in options available to decarbonize the residential sector highlighting the necessity to act now.^3^^,^^4^^,^^5^^,^^6^ Decarbonizing heat in particular is often perceived as a daunting task since natural gas serves between 60 and 80% of the domestic heat sector in countries like the UK, the Netherlands and United States with high consumer satisfaction.^7^^,^^8^ By 2019, the UK managed to reduce its greenhouse gas (GHG) emissions by 36% compared to 2008 levels, driven by power sector emissions, which fell by 67%.^9^ While there has been steady progress in decarbonizing the power sector, mostly through deploying renewable energy and replacing coal with gas generation, decarbonizing the heat sector remains an unsolved riddle on the energy agenda. Emissions from residential buildings, which account for 14% of UK emissions in 2021, only fell by 17% over 1990–2020. So to reach zero, emissions reductions will need to increase six times as quickly as they have historically.^10^ Moreover, the Russian invasion of Ukraine has heightened attention to energy security and to the price volatility associated with reliance on gas for home heating. The carbon intensity of the heat sector is driven by the incumbent gas-dominated system which serves almost 80% of demand across residential, commercial and industrial sectors.^10^ Given the high operational efficiency and low cost of the gas system, decarbonizing the heat sector will require judicious decision making and high levels of policy intervention.

Full electrification of the heat sector and replacing natural gas with hydrogen and hybrid systems including district heat networks and cogeneration technologies are the main heat decarbonization pathways being advanced.^11^^,^^12^^,^^13^^,^^14^^,^^15^ Each pathway is characterized by distinct trade-offs and a high degree of uncertainty related to the end cost for heating as well as the efficiency and security of the resulting low-carbon system. To date, most research has examined the problem of heat decarbonization by considering aggregate representations of the spatial and temporal scales of the problem on a national level^16^ and the impact of operational and security constraints on the resulting energy infrastructure has been neglected. In its 2018 overview publication, the UK Department of Business, Energy & Industrial Strategy (BEIS), outlined developments and policy initiatives on the topic of heat decarbonization arguing that no single technology can prevail as dominant so far.^10^ Electrification, biomass and hydrogen were advanced as the three main pathways whereas in its 2013 publication electrification was proposed as the dominant pathway.^17^ Concerns over electrification often center on expected pressures on the power grid and the perceived need for a very significant increase in generation capacity by as much as 3-fold.^18^

We use modeling and optimization to elucidate the implications of decarbonizing the domestic heat sector in Great Britain (GB) through electrification and present for the first time a high-resolution regional analysis. A key contribution of our study is the derivation and modeling of region-specific domestic heat demand profiles across the 13 local distribution zones (LDZs) of the GB gas network. The goal of our study is 2-fold: (1) provide a systems-based examination on the implications of electrifying domestic heat in GB and (2) identify the factors that act as barriers and enablers in the cost-optimal pathways for domestic heat decarbonisation.

### Analyzing the domestic heat sector in GB

**Figure 1:**
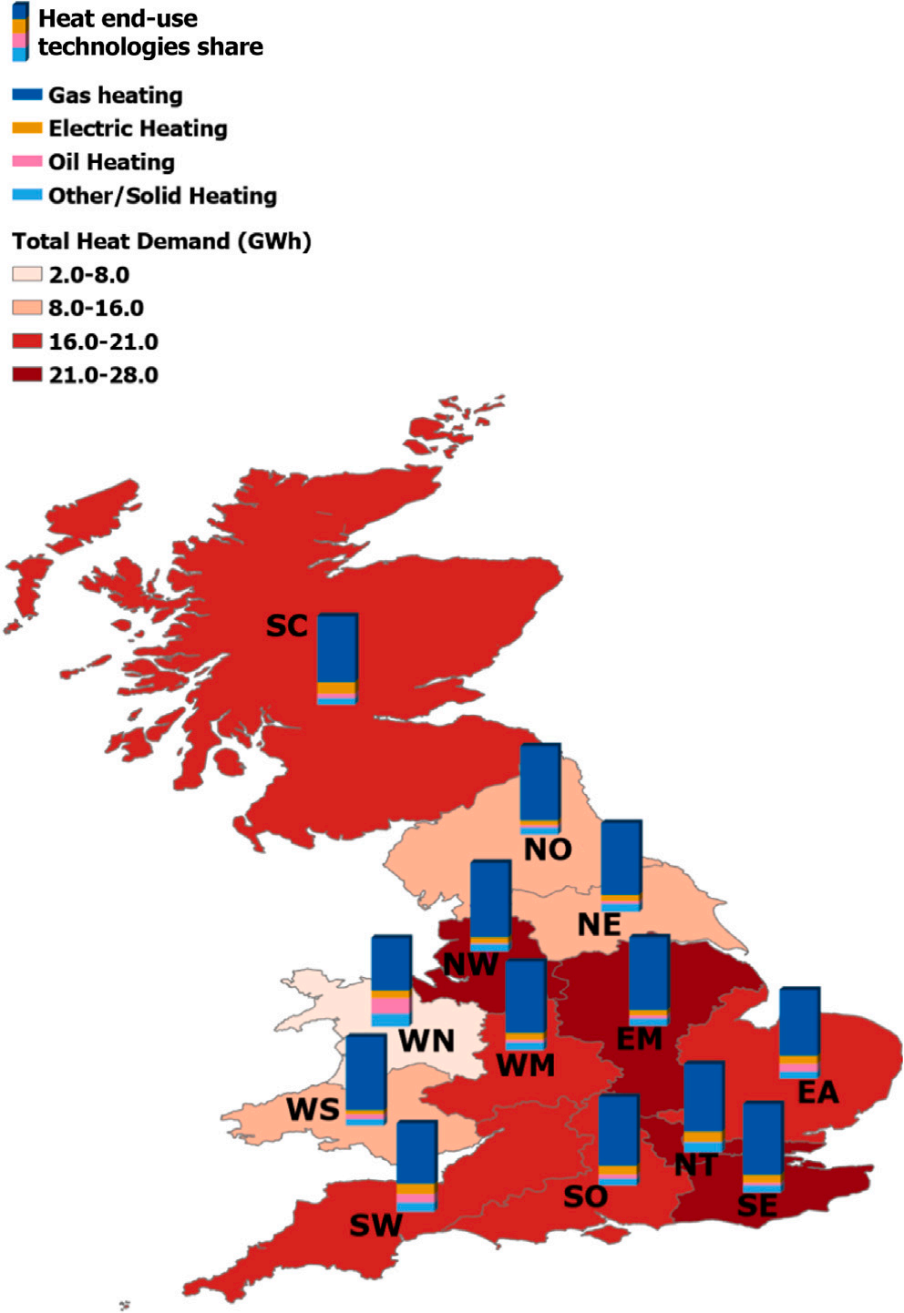
Regional analysis of the share of installed heating systems across the different regions in GB along with the total regional heat demand (MWh, given in red shading East Anglia (EA), East Midlands (EM), North East (NE), North (NO), North Thames (NT), North West (NW), Scotland (SC), South East (SE), South (SO), South West (SW), West Midlands (WM), Wales North (WN), Wales South (WS).

Almost 80% of British households are connected to the gas grid while the remaining 20%, amounting to approximately 3.5 million households, are off-grid. Of the off-gas grid properties, 36.6% use some form of electric heating, 40.8% utilize solid fuels (e.g., biomass and coal) and 22.4% use oil burners.^19^ Other forms of heating technologies such as district heat networks, ground-source heat pumps (GSHPs), air-source heat pumps (ASHPs) and micro combined heat and power systems (micro-CHP), all of which constitute viable options but to date have experienced limited adoption and taken together represent less than 2.6% of domestic heating systems in the UK.^10^ In terms of incumbency, as indicated by Figure 1, the regions in the North of England (NO, NE, and NW) have the lowest share of electric heating technologies while the South of England (SW, SE, and NT) have the largest share. Interestingly in Wales, there is a significant divergence between the two regions, WS and WN, which can be attributed to 50% of WN properties not being connected to the gas grid and hence there are large shares of both electric and oil heating. In Scotland, more than 26% of domestic properties are not connected to the gas grid (13.5% electric heating and 13% oil/solid fuel).

### Regional domestic heat demand in GB

Deciphering the impact of heat electrification in GB is explicitly dependent on the underlying heat demand characteristics of the different regions. Compared to non-heat-related electricity loads, heat demand is both highly volatile (in terms of ramp-rate changes) and seasonal. To date, one key challenge has been the lack of heat demand data at high temporal and spatial resolution. In GB, gas consumption data over the 13 different LDZs is only publicly available by National Grid with daily resolution^20^ which impedes any analysis on the operational implications for the power system. Only a handful of studies make use of (half) hourly heat demand data^16^^,^^21^^,^^22^ to examine heat decarbonization in GB. However the aforementioned time series suffer from three shortcomings: (1) they are based on a limited number of smart-meter trials,^23^^,^^24^ thus generalizing to the actual building stock can be problematic, (2) the same demand profiles are applied uniformly across regions, thus neglecting differing socioeconomic and climactic factors that affect consumer behavior and (3) scaling-up individual profiles on a regional scale is subject to several assumptions about after diversity maximum demand (ADMD) which directly affects the sizing and performance of the resulting energy system infrastructure. ADMD accounts for non-coincident factors that explain the phenomenon under which the actual observed demand from a collection of households is less than the direct summation of their respective loads.^25^

**Figure 2:**
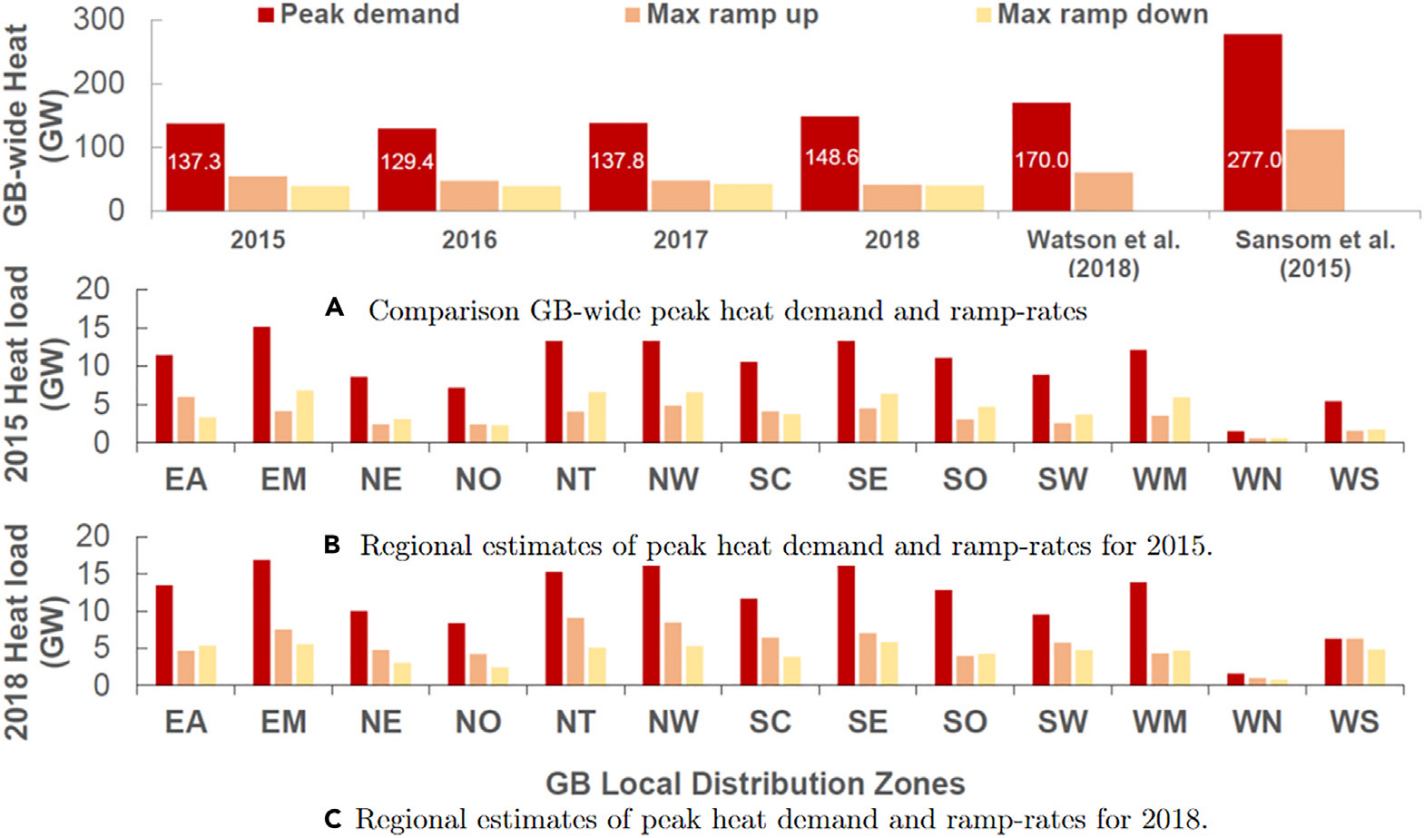
Comparison of estimated GB heat demand characteristics for different years and with previous estimates (A) Comparison GB-wide peak heat demand and ramp-rates. (B) Regional estimates of peak heat demand and ramp-rates for 2015. (C) Regional estimates of peak heat demand and ramp-rates for 2018.

To this end, we obtained hourly gas consumption data from the exit points of the LDZs over a number of years from all British gas network operators (GNOs) and analyzed time series to develop hourly and region-specific domestic heat demand data. Further details on the data and methodology are given in the Methods section while a detailed analysis of the heat demand correlation to ambient temperature is provided in Note S2, where a per household annual heat demand summary is also presented. Noting that previously estimated values cover the year 2010 which based on BEIS’ official heating degree days (HDDs) analysis was 20% colder than 2018 and 22% than 2015,^26^ our analysis indicates that the domestic peak heat demand in GB can be up to 149 GW which is up to 53% less than previously widely cited values^27^^,^^28^ and compared to recent studies^29^ the reduction in the estimated peak is a further 14%, while the maximum hourly ramp rate in heat demand was found to be 54 GWh which is 10% less than previous estimates. The importance of taking into account actual regional heat demand characteristics is underscored by Figures 2A–2C, which highlight the variations in peak load and maximum ramp rate.

**Figure 3:**
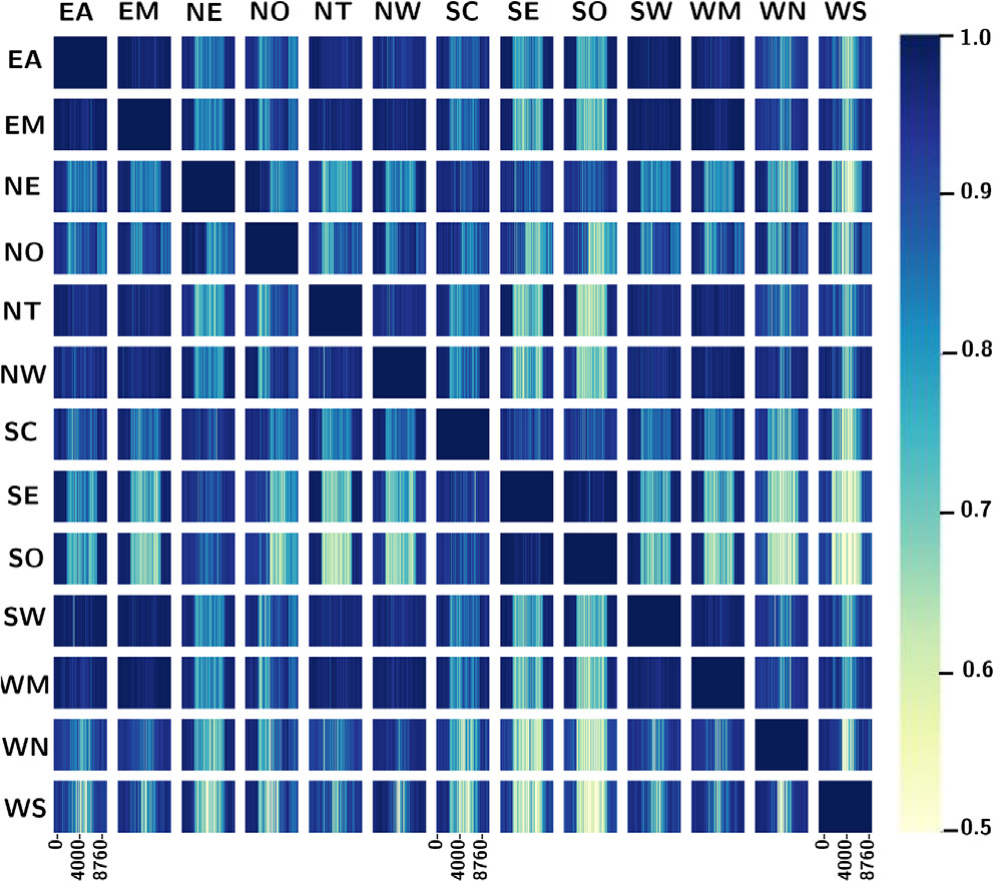
Temporal and spatial heatmap of sliding window correlation analysis Each square block represents the temporal synchronicity of heat demand pattern between two regions. Darker areas indicate synchronous demand pattern while light areas indicate greater divergence.

Given the striking divergence from previously estimated heat loads, a sliding-window correlation analysis across the spatial and temporal scales was performed to visualize and quantify the importance of region-specific and actual heat demand profiles. As shown in Figure 3, overall heat demand is as expected strongly dependent on ambient temperature and hence we identify high coincidence in neighboring regions. Nonetheless, in comparing regions that are not spatially proximal, e.g., NW and SE, we see that their temporal heatmap is not uniform (across the x axis of each square) and hence even though both regions exhibit high heat demand peaks (Figure 3), the overall peak diverges due to non-coincidence. Of course, non-temperature phenomena, such as social factors or differences in building stock, can affect hourly heat demand, which can be seen when comparing the heat demand synchronicity in adjacent regions such as SE and NT, WN and WS, or NO and NE. These neighbouring-region pairs experience similar hourly temperature patterns so it would be natural to expect high levels of correlation across temporal scales. Instead, we observe significant variation on an hourly basis, which in turn can explain the reduction in heat peak and ramping estimates.

The differences are revealed more clearly, once we perform a regression analysis on a daily and hourly basis. After aggregating our historical data on a day-by-day basis, a segmented linear regression explains well the dependence of heat demand on temperature. However, replicating the analysis using a finer (hourly) temporal scale, reveals that the relationship between heat demand and temperature is nonlinear, indicating the importance of other factors aside from temperature (see Note S2). Notwithstanding, heat demand patterns can be more accurately explained on an hourly and sub-hourly basis by taking into account factors such as occupancy schedules and thermostat setbacks, solar gains, and non-temperature weather conditions.

In terms of regional diversity, the largest regions such as North West (NW), East Midlands (EM), NT, and SE exhibit peak demand of up to 19 GWh for 2018, which represents the extreme year in our analysis, whereas the smallest regions such as the two Welsh LDZs have peak demand of less than 5 GWh. Focusing on the time series for 2018, while the GB-wide peak domestic heat demand occurs on 1 March between 18:00 and 19:00, this is not the case in every region. Specifically, while the peak is indeed synchronized for the English regions EM, NE, NO, NW, SW, and WM; in the two Welsh regions, peak demand was on the same day but earlier—07:00–08:00 for WS and 17:00–18:00 for WN, and similarly the southern and eastern English regions (SE, SO, and EA) exhibited a morning peak between 07:00 and 08:00. By contrast, the NT and SC regions’ peak was the day before (28 February) between 18:00 and 19:00. Such asynchronous regional responses, highlight the necessity of using regional data to evaluate decarbonization strategies, since peaks can occurs at distinctly different days and/or times (see Note S2).

### Scenarios and system description

To accomplish our twin goals of providing a systems-based study of GB heat electrification and drivers of cost-optimal pathways, we propose a new spatially explicit multi-period mixed integer model (OPHELIA) that simultaneously optimizes capacity and transmission expansion (on a five-year basis) as well as operational decisions (on an hourly basis). Final electricity demand is endogenously computed and is divided into heat-driven and non-heat related demand. In line with a recent study commissioned by the Committee on Climate Change,^28^ we assume that the effects of population growth and improved welfare will be counterbalanced by energy efficiency improvements in the buildings, which result in reduced demand per capita, future heat demand was based on 2015 and 2018 values respectively. For non-heat electricity demand, we follow the projections of the GB system operator^30^ and consider annual energy requirements of 307TWh (excluding losses) and GB-wide peak demand of 57 GW (excluding losses).

Electrification of transport is not considered as part of the study because of our aim to decipher the impact of the heat demand seasonality and variability on the future power sector. A detailed overview of the mathematical formulation of OPHELIA along with the list of assumptions is provided in Note S3. Additional information on the derivation of regional electricity demand as well as the techno economic data are provided in Note S4.

To analyze the impact of different system assumptions on cost-optimal electrification we consider four main scenarios. In our base scenario “Elec”, we assume heat is electrified by deploying ASHPs that are fully flexible and can be used in conjunction with thermal energy storage (TES). Scenario “ASHPFlex” differs from “Elec” in that ASHPs are considered to have constrained flexibility and can only ramp-up/ramp-down up to 70% of their nameplate capacity.^31^ To quantify the role of TES in electrifying heat, we study the scenario “NoTES” in which case electrification is only achieved through ASHPs. Despite the fact that demand side response (DSR) is not considered for domestic heat loads, the aforementioned cases can be used to examine the flexibility offered by load shifting through TES. This modeling choice is also in line with a 2022 report commissioned by the Greater London Authority^32^ in which the potential for DSR for heating was found to be limited. The “NoICPeak” scenario is similar to our base “Elec” scenario but no power imports or exports are allowed through interconnection on the peak heat day. Finally, to assess the incremental effect of heat electrification, we consider a “NoHeat” scenario where the power sector is fully decarbonized by mid-century but not heat.

As end-use heating technologies we consider: (1) ASHPs, (2) TES, and (3) gas boilers. Although GSHPs and resistive heaters (RH) are also suitable electrification technologies, the latter were not considered due to their inferior performance compared to ASHPs and the former would require information on the regional building stock and space availability, two factors that are out of the scope of the present study. As TES, we consider generic insulated hot water tanks. Finally, for the off-gas grid properties we consider full electrification of all related heat requirements (i.e., ASHP adoption).

## Results and discussion

### System-wide implications of 100% heat electrification

**Figure 4:**
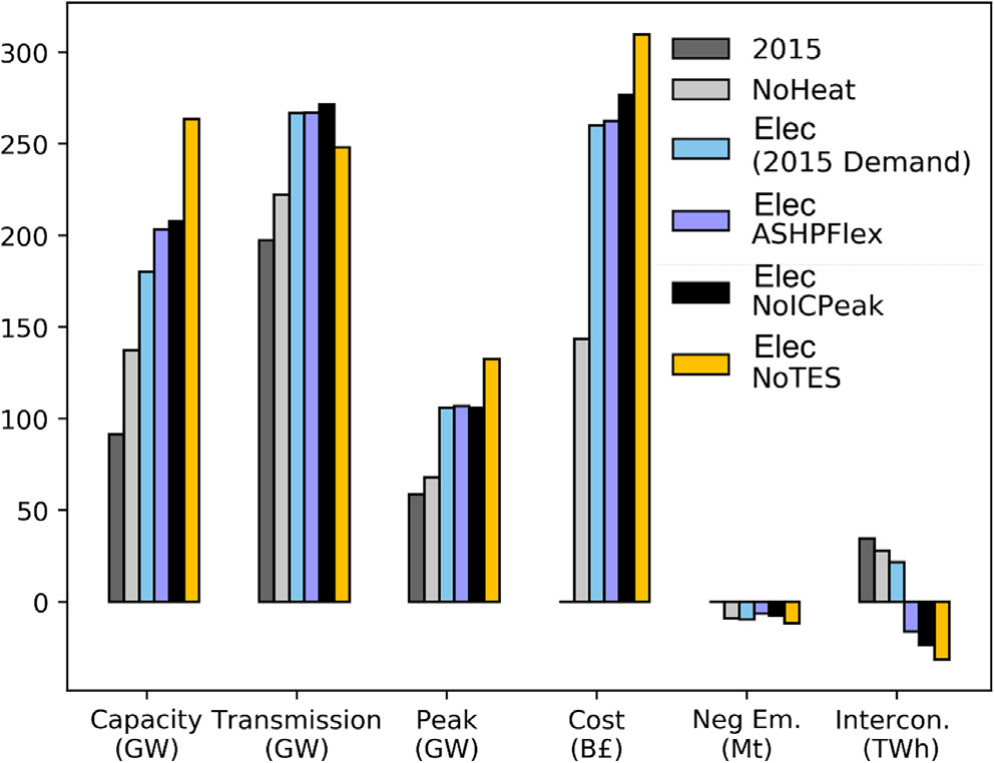
Overview of system-wide impact of domestic heat electrification under different scenarios NoHeat: Scenario of only power sector decarbonization. Elec: Base scenario of power and heat decarbonization through heat electrification. In this scenario, ASHPs are assumed to have full flexibility and TES can be deployed. ASHPflex: Same as the Elec scenario, but ASHPs can ramp up/down only up to 70% of nameplate capacity each hour. NoICPeak: Same as the Elec scenario but no interconnection is allowed on the peak heat demand day. NoTes: Same as Elec scenario, but no deployment of TES is considered.

Overall, comparing the “NoHeat” and “Elec” scenarios, for 2015 heat demand, electrification could be achieved through a 33% increase in generation capacity and 18% increase in transmission capacity between regions to meet a system-wide peak demand that increases by 56% (106 GW vs. 68 GW). An additional £100bn in capital investments would be needed to deliver sufficient power generation capacity to ensure system security and adequacy under the increasingly seasonal load that a future power sector would have to face under a 100% electrification scenario. A summary of the system-wide changes for the different scenarios is found in Figure 4.

These differences intensify when system planning is performed under more extreme years, such as 2018, when heat demand data capture the temperature variability of a European cold wave (the so-called “Beast from the East” plus Storm Emma). Specifically on 1 March 2018, gas demand in the distribution networks reached nearly 360 mcm which was higher than the 1-in-20 peak demand forecast that was published as part of GB’s gas transmission operator’s Ten Year Statement.^33^ In “Elec2018”, the total system peak reaches 113 GW (a 67% increase compared to the “NoHeat” scenario) while a further 10% increase in total system cost (TSC) is observed compared to the “Elec2015” scenario, with 28% generation capacity (mostly in the form of Nuclear, bioenergy with carbon capture and storage [BECCS] and offshore wind) and 4% additional investments in transmission capacity.

Considering only power sector decarbonization, as shown by Table 1, the optimized generation capacity is dominated by renewable generation technologies (52%), with onshore wind accounting for 25% of the capacity mix. The deployment of combined cycle gas turbines with post combustion carbon capture and storage (CCGT-CCS) begins in 2033 and steadily grows to reach 21.5 GW capacity by mid-century, while 1.5 GW of BECCS is deployed to provide negative emissions. Relative to our central scenario (“Elec”) using 2015 (2018) heat demand, the most notable changes are the increase in Nuclear capacity by a factor of 2.4 (2.8) and in offshore wind capacity by a factor of 3.3 (3.6). For the “Elec2018” scenario, aside from those changes, the value of CCS is further highlighted as a source of flexibility in extreme years—BECCS capacity doubles to 3 GW and CCGT-CCS capacity increases by 1 GW. Electrification of heat also impacts the timing and spatial deployment of CCS technologies, with investments in CCGT-CCS technologies taking place a half-decade earlier for the “Elec” scenario compared to the “NoHeat” scenario. As an illustration of differences on a regional level, 1 GW of CCGT-CCS is deployed in the NT region in the “NoHeat” scenario, but when heat electrification is taken into account, final capacity in the region is increased by a factor of 4.5. As seen in Figure 3B, this can be attributed to the high heat demand peak in NT.

**Table 1:** Impact of heat electrification on capacity and generation by 2050 for different scenarios

	NoHeat	Elec2015	Elec2018	Elec NoTES (2015 heat data)	Elec NoICPeak (2015 heat data)
Nuclear (GW)	6	14.4	16.8	23.4	15.6
CCGT (GW)	18	18.5	18.5	20.5	18.5
CCGTCCS (GW)	19	21.5	21.5	23.5	21.5
Biomass (GW)	7.7	11.2	12.9	13.3	13
BECCS (GW)	1.5	1.5	3	7	4
WindOn (GW)	35	39.6	50	55	46.7
WindOff (GW)	12	38.7	41.9	44.5	42.5
Solar (GW)	26.2	19.3	38.4	45	28
GridStorage (GW)	16	17	19	32.9	20
Total Capacity(GW)	141.4	181.7	222	265.1	210
Total Generation (TWh)	353	530	593	570	563

Overall, as Table 1 indicates, a potential 100% electrification of heat would require an almost 2-fold increase of firm generation capacity (nuclear, biomass) in the best case (“Elec2015”) while in the worst case (“NoTES”) a direct electrification with limited flexibility would require a 3-fold increase. Indeed, TES capacity provides an important additional source of flexibility and this becomes more obvious when focusing on the off-gas grid areas in which for the “Elec2015” and “Elec2018” scenarios a total capacity of 23 GW and 28 GW, respectively, is installed while, surprisingly, in the “NoICPeak” scenario, the installed capacity is only 20 GW. For grid-connected properties, TES is in-stalled in tandem with ASHPs following a similar trend as that of off-gas grid properties. From a renewable generation perspective, heat electrification appears to favor investments in wind rather than solar generation due to issues related to synchronicity on the availability of solar vs. heat demand patterns. Finally, another interesting aspect is the potential competition between grid-level storage technologies and TES as either can be used for absorbing RES intermittent generation (cf. “Elec”, “NoTES” scenarios).

### Regional drivers and adoption rates

**Figure 5:**
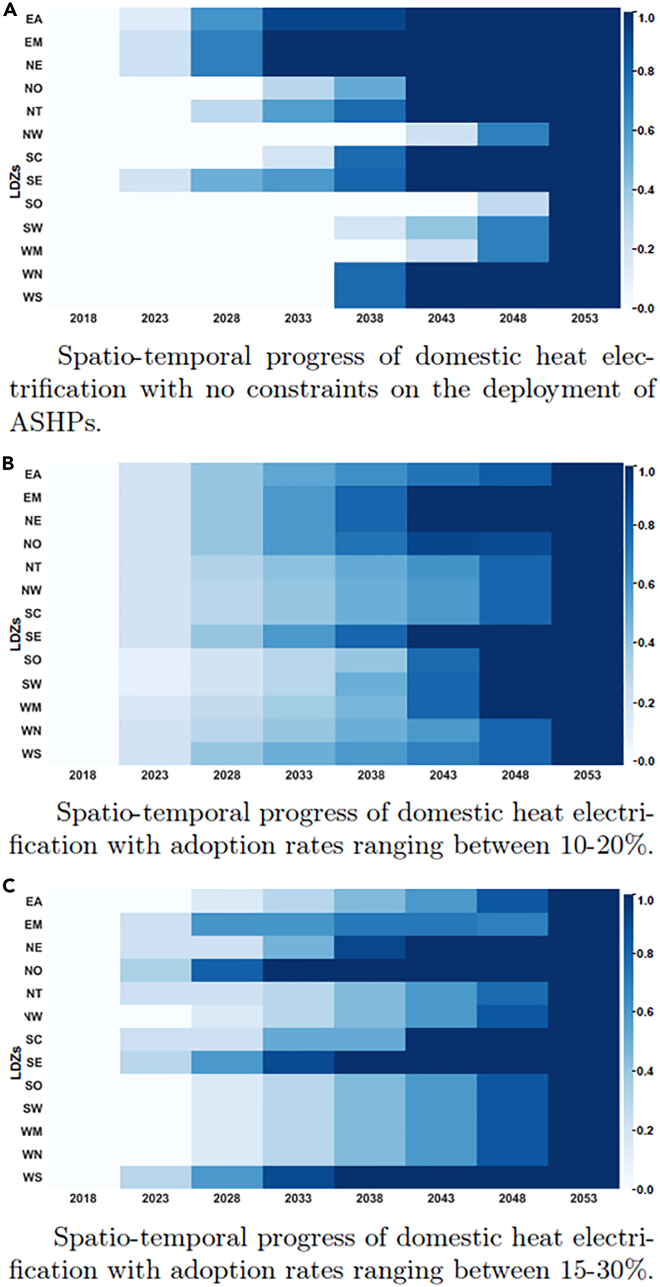
Domestic heat electrification rates under the “Elec” scenario of on-gas grid properties across different regions in GB, following different scenarios for ASHP adoption rate Progressively darker shades indicate a higher percentage of electrified properties in each region at each time step. (A) Spatio-temporal progress of domestic heat electrification with no constraints on the deployment of ASHPs. (B) Spatio-temporal progress of domestic heat electrification with adoption rates ranging between 10 and 20%. (C) Spatio-temporal progress of domestic heat electrification with adoption rates ranging between 15 and 30%.

In this section, we delve into the spatiotemporal evolution of the GB energy system toward 100% heat electrification. As indicated by Figure 5A, in our base scenarios where no adoption rates constraints are imposed, there is great disparity in regional electrification rates. Eastern regions (EA, EM, NE, and SE) appear to be early and steady adopters throughout the planning horizon whereas other regions such as NW, WM, SO, and Wales only become electrified toward the end of the time horizon based on very high adoption rates. Detailed discussion of the regional capacity allocation results is presented in Note S5, which can explain the regional variation. Focusing on the Welsh regions (WN and WS), for example, we observe that in the unconstrained case, they are electrified concurrently with increased installation of wind power generation and grid-level storage as can be observed from the regional capacity allocation results presented in Note S5. When adoption rate considerations are not taken into account, the eastern regions can exhibit electrification rates ranging from 35% to 56% over a 5-year period, which far exceeds any previous electrification rollout, and so might be viewed as unrealistic.^34^ To this end, and to shed light on key barriers/enablers for early electrification, we explore different scenarios while imposing a requirement that regional adoption rates lie within either: (1) 10%–20% or (2) 15%–30% over a 5-year period. The results of these runs are presented in Figures 5B and 5C. It is interesting to note that the 10–20% adoption rate case reflects the UK’s government’s ambition to install 600,000 heat pumps annually from 2028 following its recent “Ten point plan for a green industrial revolution.”^35^

To guide our analysis, let us focus on SE and SO, which, though neighboring regions, follow completely different paths to electrification, as seen in Figures 5A–5C. These two regions are also interesting because, apart from similar temperature conditions (which affect ASHP performance), they also exhibit very similar heat demand patterns as shown in Figure 3. In the base scenarios, the SO region is a net importing region while SE is a net exporter, mostly to the NT region. When adoption rates are constrained, however, power flows are reversed in both regions. For example, when adoption rates are constrained, SE exports on a net basis to SO but is a net importer from NT, which, in turn, reduces the electrification rate in NT. The general trend indicates an increase in firm generation, mostly through more nuclear and some biomass power plants, with subsequent reduction in the regional share of intermittent renewable energy sources. This trend is particularly apparent during the early periods (2023–2038) before the uptake of CCS technologies (BECCS or CCGT-CCS). For instance, comparing generation capacities in SO during 2028 we identify an increase of 0.7 GW in biomass capacity and 1.5 GW in grid-level storage capacity in both constrained scenarios versus the unconstrained case. The same trend is identified in the SW region where solar capacity declines by 1 GW in the case of 10–20% and by 2 GW when 15–30% rates are imposed. In both scenarios 2.4 GW of nuclear capacity is deployed in SW in 2028 whereas in the unconstrained case no nuclear capacity is installed. By contrast, 3 GW of additional nuclear is expected in EA by 2033 in the unconstrained case but when adoption rates are constrained, additional installed capacity reduces to 1.8 GW and 1.2 GW for the 10–20% and 15–30% adoption rate scenarios respectively. Nonetheless, overall RES capacity does not decline in order to meet the decarbonization targets but instead is complemented in regions with investment in peaker plants (CCGTs and OCGTs) and grid storage. That is the case for NT, where full heat decarbonization is delayed by a decade in the constrained adoption rate scenario, but both solar power plant capacity (+3 GW) and CCGT (+3 GW) increase in 2033. Similar insights are derived by examining the NO and NW regions, where firm generation and grid-level storage both increase when adoption rates are constrained with an additional 600 MW of nuclear power invested in NW in 2033 and an extra 500 MW of CCGT-CCS power introduced in the NO region (see Note S5).

### The cost of reduced flexibility in heat electrification

Sources of flexibility become crucial in alleviating the challenges of direct electrification of heat. In particular, we identify and study three different factors: (1) the use of TES (NoTES), (2) the operational flexibility of ASHPs (ASHPflex), and (3) the ability to import/export power during peak heat demand days (NoICPeak). A decarbonized system without TES deployment requires a total capacity of almost twice that needed for decarbonizing the power sector alone due to the doubling in the overall peak the system has to meet (132 GW vs. 68 GW). The reduced flexibility due to the absence of TES results in a 35% increase in TSC and leads to asset under-utilization, with the average utilization factor for CCGT-CCS dropping to 36% in the “NoTES” from 69% (“Elec” scenario) for the case of 2018 heat demand.

In the “ASHPflex” and “NoICPeak” scenarios, generation capacity increases by 50% compared to the “NoHeat” case, while the absence of interconnection during the peak heat day results in a 22% increase in cross-region transmission capacity utilized to counterbalance the lack of interconnection in coastal regions. Comparing the “Elec” scenario with the “ASHPflex” and “NOICPeak” we observe that the lack of flexibility in both cases results in the additional investment of 1.2 GW of nuclear power. Additionally, in the “NoICPeak” scenario an extra of 1 GW of BECCS is installed to counterbalance the emissions incurred by the increased utilization of CCGTs (+4.3% generation increase in the “NoICPeak” case compared to the “Elec” scenario) and satisfy the security requirements for the capacity reserve margin. In both cases, an additional £10bn and £18bn increase is reflected in the TSC while GB ends up being a net power exporter by mid-century mostly to Norway and Denmark due to the increased generation by onshore and offshore wind power plants. Nonetheless, the interconnection flows rely upon future price projections based on European Network of Transmission System Operators for Electricity’s Ten-Year Network Development Plan according to their “Global Climate Ambition” (GCA) (or deep-decarbonization) scenario.^36^

### Limitations of the study

Interpreting the results from OPHELIA (or any model) must be treated with some caution due to a number of limiting factors. First, given the vast uncertainty related to future domestic heat demand dynamics we have assumed that the shape of the hourly heat demand profiles remains the same in future years. The same holds true for the shape of the electricity demand profiles.

Regarding the presented heat demand data, a validation has not been possible as this would require a specialized smart meter project such as the 2007 Carbon Trust Micro-CHP Accelerator project^21^ or the Energy Demand Research Project (EDRP)^29^ that was carried out between 2007 and 2010 were a significant number of consumers would need to participate.

Detailed characteristics of demand such as the energy efficiency of GB’s building stock and the behavior of the residents of those buildings will affect the process of decarbonization. Changes to both electricity supply and demands for energy in the residential sector are highly dependent on policy interventions related to governmental subsidies and regulations which are highly uncertain. In light of this, expanding high-resolution mathematical models such as OPHELIA to account for such uncertainty is a necessary next step and a subject of our ongoing research.

Finally, given the scope of the study, even though decarbonization of the transport and the industry sectors will undoubtedly affect some of the decisions related to the decarbonization of the domestic heat sector we have not included such decisions as part of OPHELIA to date.

### Conclusions

Full electrification of heat will be challenging for many reasons apart from the demands placed on the electricity system. Reaching high sustained adoption rates will require significant government incentives and will involve engaging not just early adopters but will require a shift away from gas in large commercial establishments and among late adopters and laggard domestic consumers, who will be skeptical of the technology and/or daunted by the capital expense.

Current UK government policy to incentivize heat pumps badly lags behind other European countries and the government’s own targets. As evidence that such deployment is possible with more effective incentives, many European countries (Italy, France, Poland, etc) have recently seen a dramatic increase in installations. Indeed, in 2022, Italy and France, which have building stock comparable in size to that of the UK sold 513k and 621k heat pumps respectively, which would be in line with the UK government targets for 2028.

The economics of maintaining the existing gas infrastructure in the transition to full electrification with ever-smaller volumes of gas is also challenging. Moreover, there are numerous important questions that remain such as how to maintain gas in the system for hybrid heat pumps and what the basis should be for sizing a fully electrified system. From a carbon reduction perspective in the short run such complications may not be insurmountable, but in the long run they can lead to deadlocks due to the mixed market signals being sent, e.g., on the future of natural gas, as well as undermining a smooth policy-driven transition to low-carbon heat. Future research should build on spatially explicit and multi-period modeling to explore integrated capacity expansion planning and operational optimization of the integrated heat and power system, particularly in the context of the role negative emissions might play in decarbonizing the heat sector. While there is no silver bullet to decarbonize heat, we have shown in the present study that electrification of heat in conjunction with smart operation of TES constitute a viable candidate without needing unreasonably rapid growth in overall system capacity although deploying TES at scale is challenging.^37^ The additional capacity needed would increase by roughly 40% without TES. This reinforces Schill^38^ who highlights the importance of deploying TES as a seasonal balancing option for electrified heating systems. Although we have demonstrated that electrification is not as daunting as some have claimed, this is only one part of the heat puzzle and the potential role for hydrogen and biomass need to be investigated in similar detail so as to decipher the underlying synergies and this constitutes ongoing research within our group.

## STAR★Methods

### Key resources table

**Table:** 

REAGENT or RESOURCE	SOURCE	IDENTIFIER
**Software and algorithms**		

General Algebraic Modeling System (GAMS) version 42.1.0	GAMS Development Corporation	www.gams.com
Python version 3.8.10	Python Software Foundation	www.python.org

### Resource availability

Further information and requests for resources should be directed to and will be fulfilled by the corresponding authors.

#### Lead Contact

Further information and requests for resources should be directed to and will be fulfilled by the Lead Contact, David M. Reiner (d.reiner@jbs.cam.ac.uk).

#### Materials availability

This study did not generate new unique reagents.

### Method details

#### OPtimising heat ELectrificatiOn regIonaL strAtegies (OPHELIA) model description

OPHELIA simultaneously minimizes the power and heat system costs to satisfy the related loads on an hourly basis subject to technical constraints for evaluating the impact of domestic heat electrification on the power and gas systems in GB. It is a spatially explicit multi-period model where GB is discretized into the 13 LDZs of the gas network. Given existing and projected power generation capacities in GB, the model optimizes: (i) new power generation and storage capacity locations; (ii) hourly dispatch decisions; (iii) power transmission flows within the considered GB regions; (iv) interconnection flows with third countries; (v) hourly upward and downward reserve requirements and commitment; (vi) heat generation and storage capacity investments and location; (vii) hourly heat generation and storage operational decisions. For the representation of renewable energy sources, we collect hourly availability data as provided by the renawables.ninja platform.^39^ The hourly availability reflects the percentage of the installed nameplate capacity that would be generated at a given hour. To capture the variability in RES availability within each region, for solar and onshore wind we sample different spatial intraregional availability and the average of those is used as the final regional availability factor, while for the case of offshore wind generation points were considered up to 50 km from the shore. While more detailed representations of RES have been presented in recent studies,^40^ we opt for this approach as our main focus here is the impact of heat electrification and not the integration of renewables in the grid, although our model can readily consider such detailed cases as input data. In terms of reserve requirements, we model upward short-term operating reserve as a function of the forecasting errors in wind generation, electricity demand and the capacity of the largest generator to simulate N-1 security criteria considerations.^40^ Downward reserve requirements are modeled as a percentage of the upward requirements.^41^ Distribution losses are modeled as a percentage of the resulting regional demand, while transmission and interconnection losses are endogenously calculated as proportional to the transmitted power and distance between the different regions.^42^ Transmission corridors between different regions is modeled following the transshipment models conventions which does not account for Kirchhoff’s voltage law.^43^ One strong point of OPHELIA is the high-fidelity regional demand considerations across the different LDZs. To date, in past studies of heat decarbonization, models have employed the same hourly heat demand patterns and a limited number of representative days when regional decarbonization strategies are examined.^44^ Moreover, we differentiate between emissions reduction requirements for the heat and the power sectors to enable the examination of sector-specific budgets and their impact on heat decarbonization policies. The overall model is formulated as a mixed integer linear program and is implemented in General Algebraic Modeling System and AIMMS. A more detailed description of the model’s data, equations and key assumptions can be found in Notes S3–S4.

#### Deriving regional domestic heat demand data

Understanding and preserving the spatial and temporal variations on heat demand is vital for deriving realistic decarbonization insights and strategies. In principle, heat demand profiles are determined by a range of aspects such as behavioral, building stock and temperature conditions. A primary concern regarding the decarbonization of heat through electrification is the resulting load variability that the grid operator would face. To this end, a limited amount of works have been presented in the literature that employ half-hourly/hourly heat demand profiles.^16^^,^^21^ The shortcoming of these previous studies is that the derived heat demand profiles come from either a 2007 Carbon Trust Micro-CHP Accelerator project with 71 domestic buildings^21^ or from the EDRP that was carried out between 2007 and 2010 with around 6000 participants.^29^ While these datasets and the resulting heat demand profiles constitute a significant step in the desired direction, using them to evaluate the impact of decarbonization in a spatial manner for the UK runs into difficulties because of the limited representation of the regional characteristics of heat as well as the end regional heat load is subject to after diversity peak demand considerations which are key in designing the future grid. To this end, in the present work we employ regional hourly gas demand data as a proxy. These novel empirical data were collected from each of the GB Gas Distribution Network Operators (DNOs) and includes all hourly gas demand data for four years from 2015 through 2018 inclusive. In these time series, gas demand comprises daily metered (DM) demand (associated with large industrial premises) and non-daily metered (NDM) demand (associated with domestic, commercial and medium sized industrial premises). The reader interested in the specific definitions of these components in referred to National Grid’s methodology^45^ for a comprehensive review. To then derive the related domestic demand from the time series the following methodology was devised by employing gas standard load procedures by German Federation of the Gas and Water Industry (BGW)^46^ as well as the German Association of Local Utilities (VKU).^47^ Using their methodology, characteristic hourly and temperature-dependent gas demand profiles are presented for a range of different domestic, industrial and commercial units. In conjunction with these profiles, regional hourly temperature data,^39^ sub-national gas consumption data from BEIS (that also non-gas properties using solid using fuels for heat) in the different regions across GB^48^ were employed. Domestic demand is derived then as follows. First, using the gas standard load profiles the DM demand as reported in^46^^,^^47^ is scaled down on an hourly basis for all the LDZs. The resulting hourly DM demand was subtracted from the DNO time series leaving gas demand related to NDM customers. Then, using sub-national gas consumption data statistics about regional domestic and non-domestic percentages together with the master temperature-dependent profiles for non-domestic customers we scale down on an hourly basis the NDM consumption data available from.^49^ By subtracting the hourly non-domestic NDM component together with the related DM component from the original time series the domestic hourly gas consumption demand in the different LDZs is retrieved. Finally, for the cases where negative values were encountered in the final time series we interpolated between the neighboring data points to preserve continuity. It should be noted that for the Welsh regions (WN, WS) we employed existing regional domestic half-hourly data for heat demand^50^ because of complications in accounting for different gas flows in those regions. Finally, to derive non-electric heat demand for the proportion of off-gas grid properties within each region, we use the data about household heating technologies as presented by^19^ and assume an average of 90% efficiency for both biomass and oil burners. Further assumptions that are employed for the derivation of regional heat demand profiles include: (i) the gas-heated domestic demand is taken as representative for the whole building stock within each region and (ii) the gas boiler demand reflects directly the underlying heat demand. Further to these assumptions, with the regards to the applicability and validity of the German gas suppliers methodology we can expect to have some deviations from the ground-truth heat demand but as indicated by Ruhnau et al.^51^ using these standard profiles for the UK results in high consistency between modeled and historic behavior of the heat sector.

As shown in Figure 2A, compared to existing works on estimating the GB heat demand, on a national scale, we are in good agreement with the results of Watson et al.^29^ with 4% higher estimated peak demand (177GWh) with a larger deviation is observed at 20% for the maximum ramp up in heat demand (72GWh vs. 60GWh). Compared to Sansom et al.^24^ we derive a significantly lower peak demand (36%) and almost 45% lower maximum ramp up. With regards to the regional aspects of gas-related heat demand, as shown in Figures 2B–2C the regions EM and NT have the largest contributions to the national peak demand (around 25 GW each) while for the case of Wales the profiles derived from Knight et al.^50^ indicate a rather interesting behavior with the scale of peak demand and ramp up, ramp down requirements being quite close especially for WN as indicated by Figures 2B–2C. Apart from WN, the smallest heat peak demand is found to be in the NO region (11.3 GW) and SW region (12.4 GW) and such region-specific insights are useful when deciding regional rollout of electrifying heat as it may be preferable to electrify first those regions where their peak heat demand component is not prohibitively large.

#### Representative days selection

For tackling the computational tractability of the model’s temporal resolution we employed data clustering techniques. In particular, we employ K-Medoids clustering to agglomerate days of the year that exhibit similar patterns with respect to regional demand of electricity and gas, RES availability, interconnection prices and average temperature across the 13 LDZs. To preserve peak electricity and gas demand days, the original time series are pre-processed and these two days are excluded from the clustering and are added at the final stage. Once clustering is completed, an average day is computed and then the representative day is chosen such that it has the minimum geometric distance from that day. While clustering techniques have been widely applied to energy systems models,^52^^,^^53^ the resulting representative days are generally not placed in chronological order. However, for the case of heat decarbonization preserving the chronological order is important due to the inherent seasonality of demand. To this end, once representative days are selected for each cluster, they are organized in chronological order.

## Data Availability

• The code of OPHELIA is available from the corresponding authors upon reasonable request. • The input data for OPHELIA based on which the results presented in this article were computed are available from Zenodo: https://doi.org/10.5281/zenodo.6022815 • Any additional information is available from the corresponding authors upon reasonable request.

## Supplemental information

Document S1. Figures S1–S12, Tables S1–S10, and Notes S1–S5
