# Supplementary material for: The impact of 100% electrification of domestic heat in Great Britain
Source: iScience. 2023 Oct 18;26(11):108239. doi: 10.1016/j.isci.2023.108239 (PMC10637952; doi:10.1016/j.isci.2023.108239)
Supplement: Document S1. Figures S1–S12, Tables S1–S10, and Notes S1–S5 [file mmc1.pdf]

iScience, Volume *26*

Supplemental information

The impact of 100% electrification of domestic heat in Great Britain

Vassilis M. Charitopoulos, Mathilde Fajardy, Chi Kong Chyong, and David M. Reiner

Supplementary material: The impact of 100% electrification of domestic heat in Great Britain

Vassilis M. Charitopoulos   Mathilde Fajardy   Chi Kong Chyong

David M. Reiner

August 30, 2023

Supplementary Note 1: Overview of heat decarbonisation pathways & literature review

Heat decarbonisation would require a major transition of the energy landscape in the UK and in order to secure sustainable, low-carbon and economic heat a number of pathways have been identified. A conceptual visualisation of the individual components that comprise the different pathways is given in Fig. 1.

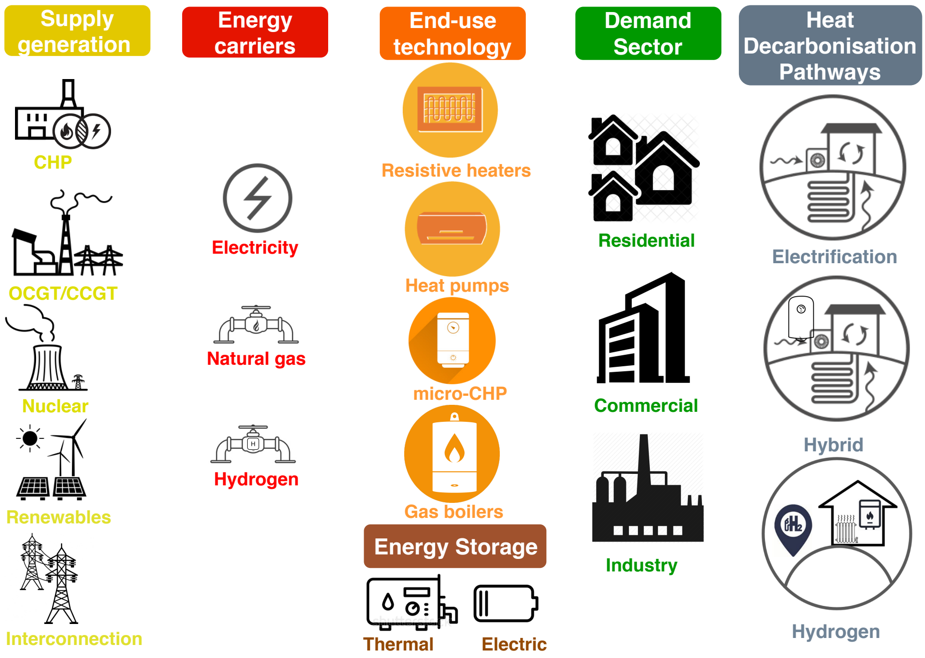

Figure 1: Whole system visualisation of decarbonisation pathways for the GB heat sector

Electrification of heat

Options for heat electrification can be categorised into two broad groups as indicated by Fig.2. In the centralised setting, heat is generated by electricity and is subsequently distributed, through district heat networks (DHN) to the end-users. Research works indicate that DHN have inherent energy storage through their pipelines [1], while additional thermal energy storage can be considered as part of their design. In the decentralised setting, electricity is converted to heat on-site

and the main options involve: heat pumps (HPs), either air-sourced (ASHPs) or ground-sourced (GSHPs), and resistive heating (RH).

The key benefits of electrifying heat is the flexibility opportunity it provides to the power system for better integration of renewable energy sources (RES) as well as the technology readiness levels of end-use options[2]. A number of studies have indicated that electricity-fuelled district heat networks coupled with thermal energy storage and demand side response measures can result in reduction of RES curtailment [3, 4]. For the decentralised scenario where individual heat pumps are deployed, the effect of heat pumps on the flexible integration within the power sector was found positive under assumptions on energy efficiency measures and thermal storage [5, 6].

Munuera et al. [7] investigated the role of energy efficiency in the electrification of heat. Utilising data from the Energy Saving Trust's heat pump field trial the effect of the building stock's energy efficiency on the correct sizing of the required HP as well as the contribution to the peak load was examined. Results from this field trial concluded that the COP for ASHPs was between 1.6-2.2 while for GSHPs 1.8-3.0 [8]. A technical feasibility study on electrifying heat in rural off-grid areas was reported in [9]. The study concludes that bigger energy savings can be realised using HPs rather than RH. Without energy efficiency improvements, 84% of homes can efficiently adopt electrified heat while the share rises to 93% with loft & wall insulation. However, the authors identified that the technical feasibility can be endangered under their 1-in-20 winter peak scenario for which only 41% to 63% (depending on the type of HP) of the homes could electrified.

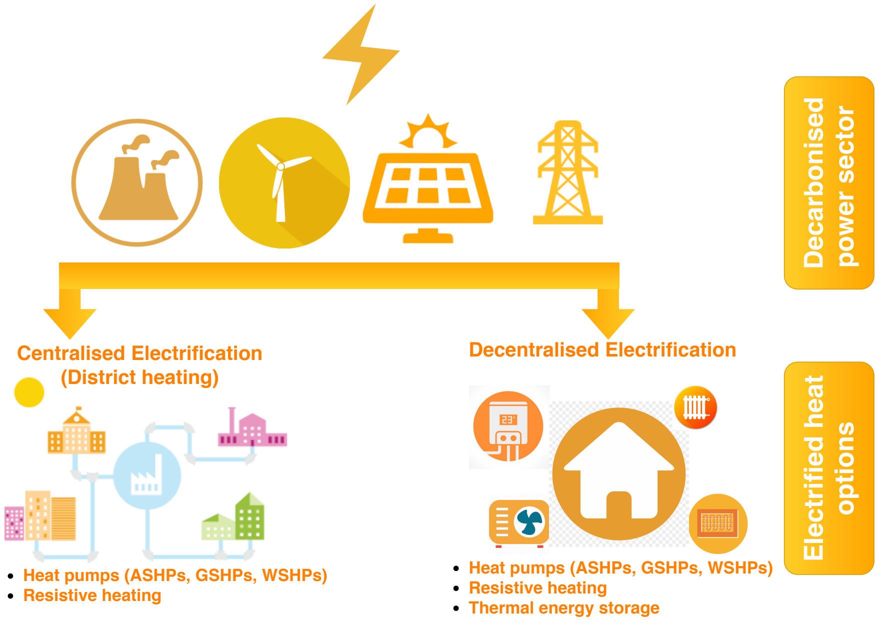

Figure 2: Categorisation of heat electrification options

Peak heat demand in the UK can reach up to four times the peak demand from the current electricity system [10]. Owing to the existing gas storage system in the UK, balancing between the demand and supply sides can be achieved without excessive capacity investments. However, for the electricity system this is not the case since instantaneous balancing is required to avoid

power outages.

Electrifying heat raises also concerns about the future of the gas infrastructure in the UK as well as the amount of investments needed. In terms of economic cost, heat electrification is characterised by high-capital and low-operational costs due to the increased efficiency of heat pumps. The main drivers from the high-capital expenditure are: (i) investments in generation capacity expansion, (ii) investments in transmission's network reinforcement and (iii) high up-front costs from the consumers for HPs [11].

Hydrogen-led heating

Among the different decarbonisation pathways in the literature, the potential of a "Hydrogen Economy" has constituted a notable alternative towards clean energy for the heat and transportation sectors in the UK [12]. and a conceptual overview of this pathway is given by Fig.3. Unlike natural gas, hydrogen does not result in GHG emissions upon combustion. Nonetheless production of hydrogen in scale is reliant on processes which result in emissions and thus its low-carbon potential is bounded with extensive investments on carbon capture and storage (CCS) [13].

In order to avoid emissions during the production process, the aforementioned technologies are considered in conjunction with CCS while for water electrolysis electricity from renewables can be utilised. Hydrogen can also be supplied as a by-product from chlor alkali and refineries processes. In the latest report on hydrogen by the Committee on Climate Change, coal gasification is not considered a viable option due to its high cost and limited emissions savings to the incumbent natural gas system. The only production route of hydrogen that does not require investment in CCS is water electrolysis using curtailed RES. The viability of such option was briefly examined and as indicated in [14] if wind curtailment reaches the level of 75TWh by 2050, then hydrogen produced by water electrolysis would only satisfy 14% of UK's domestic heat demand.

Hydrogen's decarbonisation pathway is characterised by significant uncertainties with regards to technology deployment and resulting end-costs. Shifting from natural gas to hydrogen would require considerable investments in adjusting the existing gas grid to secure safe and efficient transmission on top of replacing existing domestic end-use appliances. Dodds and Demoullin [15], interviewed specialists from industry, government and academia and conducted a simulation study on the cost-optimal conversion of the existing UK gas system to transport hydrogen. The key findings indicated that the current high-pressure transmission gas system would need to be modified to safely transport hydrogen while the low-pressure system does not need any modifications. Another issue was found to be the resulting reduced capacity and linepack storage compared to natural gas due to different energy volumetric densities of the two fuels. The future of the UK gas system was the focus of [16]. The authors using a revised version of the UK MARKAL model to compare the scenarios of injecting biomethane to the gas grid or converting it for hydrogen concluded that the cost-optimal choice is the hydrogen solution.

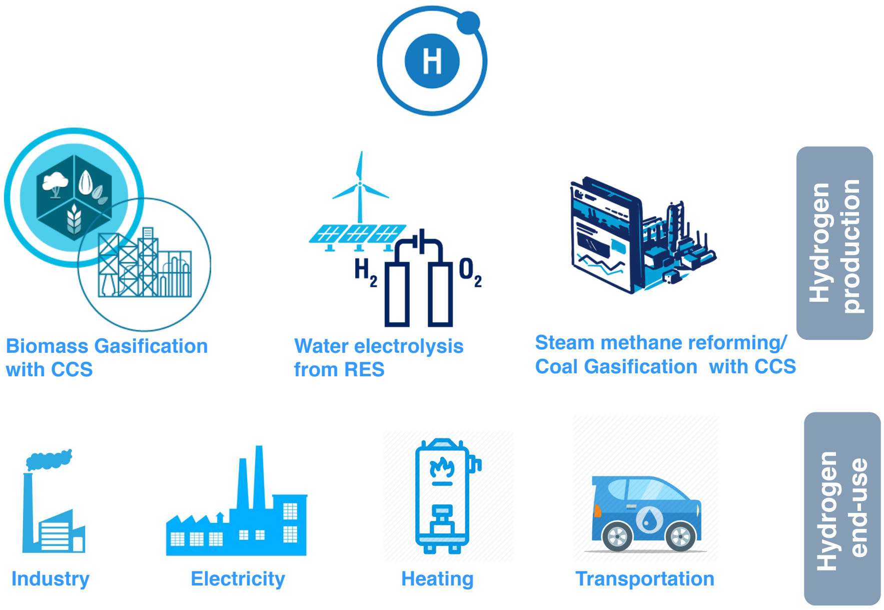

Figure 3: Conceptual representation of hydrogen economy production and end-use pathways

Existing literature on modelling of decarbonisation pathways for the heat sector

Electrification of domestic heat is likely to result in excessive electricity infrastructure expansion given that hot water and space heating account for almost 80% of the energy use of the buildings sector in Europe and 60% in the US. In the literature a variety of strategies have been proposed for the decarbonisation of heat which can be broadly categorised as follows: (i) electrification of heat, (ii) low/zero-carbon gases, e.g. hydrogen or biomethane, (iii) district heating networks that supply heat from low-carbon sources, (iv) efficiency measures and (v) behavioural change related approaches.

A spatially-explicit, multi-period MILP model was developed by Jalil-Vega and Hawkes to investigate the heat supply and network infrastructure trade-offs in the City of Bristol [17]. The authors considered both domestic and commercial demand while a number of different end-user technologies were involved including heat pumps and district heating networks. Gas, electricity and heating network infrastructure costs were also considered. The same authors later on examined the role of hydrogen towards the decarbonisation of heating [18]. They revised their model to account for hydrogen demand however infrastructure decisions were only made on a aggregate network level. In their results, heating demand was primarily satisfied using district level heat pumps with hydrogen boilers being the second technology in demand satisfaction, under a flat heat demand assumption. Quiggin and Buswell presented a model for estimating the impact of heat electrification in the UK from a demand-supply viewpoint [19]. Six published scenarios for the decarbonisation of heat were investigated and underlined the role of demand side management and heating demand reduction measures for securing supply. In [20], the impact of exploiting thermal building inertia and the effect of weather events on heat demand was studied. The authors integrated a "resistance-capacitance" representation in their energy planning model

for the case of Ireland and showed that thermal building inertia can reduce operational and capital costs for both the demand and supply side while also contribute to more flexible deployment of RES.

An analysis of the gas and electricity demand profiles in the UK is conducted identifying the variable component for heating. The authors used data available from the National Grid and examined the scenario where 30% of the NMD from gas is transferred to electrification either with resistive heating or heat pumps. The role of domestic energy efficiency, seasonal storage, biomass heating were briefly discussed.

In [21], an overview of the key technological and economic uncertainties related with the decarbonisation of heat in the UK is given. The authors provided a high-level analysis on the affect different uncertainties, such as fuel price or efficiencies, have on the final energy system costs and carbon emissions.

The impact of hybrid heating devices towards decarbonisation of heat in Ireland was studied in [22]. In this work, an investment model was formulated as linear program and the least-cost option was found to be the hybrid ASHPs-gas boilers under different scenarios on future gas prices and building energy efficiency measures.

Heinen et al. (2016) investigated the economic and operational performance of different hybrid heating technologies and concluded that the combination of gas-boilers and electric heat pumps provide significant advantages over the other options such as resistive heating and gas boilers. Heat demand density has been reported as a determining indicator for the suitability of region to benefit from distributed heat networks. McKenna and Norman (2010) investigated the potential of energy recovery from industrial heat loads for Great Britain in a spatially-explicit manner [30].

The opportunity of harnessing heat content from industrial activity in order to satisfy domestic space and water heating demand was also highlighted for the US case by the work of Rattner and Garimella [31]. Zhang et al. presented a whole-system study on the decarbonisation of heat in GB to optimally decide on the end-use heat generation system design involving heat-pumps, district heat networks (DHN) and hybrid deployment of heat-pumps with gas boilers (HP-B) [26]. The authors concluded that the HP-B pathway presents economic and operational benefits over the other alternatives, due to the lower capital cost of decommissioning existing domestic gas-boilers and the avoidance of reinforcing the electricity network because of heat peak demand. The deployment of DHN was found to be beneficial to some extent only in urban areas due to high heat density but always in conjunction with HP-B.

Bloess et al. [32] presented a literature review on the topic of heat electrification with emphasis on Europe and concluded that, up to an extent, electrifying heat together with flexible thermal storage technologies can prove to be beneficial for higher integration of renewables into the energy mix.

Qadrdan et al. presented a linear programming model with electricity dispatch consideration to study the impact of heat electrification on the GB gas and electricity system [33]. In their approach GB is represented by a single node and a one-year operation with half-hourly resolution was employed to model the impact of electrifying heat on the low- and high-pressure gas network

Table 1: Overview of modelling methods for the examination of heat decarbonisation pathways

Model	Purpose	Approach	Spatial resolution	Temporal resolution	Heat demand	Heat technologies	Unit commitment constraints
**FESA** [23]	Scenario analysis	Merit-order generation grid balancing	Single node	Hourly steps over one year	Hourly average constant heat demand	CHPs, HPs, RH	NaN
**SHED** [19]	Scenario analysis, hybrid bottom-up/top-down	Grid balancing	Single node	Hourly steps over one year	HDDs and Economy 7 hourly demand patterns	HPs, Solar thermal, CHPs	NaN
**EnergyPLAN** [24]	Exploration	Optimisation, simulation	National, regional	Hourly steps over one year	HDDs and daily cycle profiles	Biomass/ Gas Boilers, H_2 / μ-CHPs, HPs, RH, DHN	NaN
**ESME** [25]	Exploration	LP least-cost optimisation	12 UK regions	5-year steps until 2050 with 10 time slices	HDDs and hourly consumption data	Biomass/ Gas Boilers, μ-CHPs, HPs, RH, DHN	NaN
**HIT** [17]	Investment	MILP least-cost optimisation	City of Bristol, 55 regions	5-year steps until 2050 with 16 time slices	Hourly average constant heat demand	DHNs, HPs, HP-B	NaN
**IWES** [26]	Scenario analysis, investment	MILP least-cost optimisation	5 UK regions	Hourly steps over one year with 3 additional extreme days	Hourly heat demand data from [27]	HPs, DHN, HP-B	NaN
**Heinen et al.** [22]	Investment	LP least-cost optimisation	Single node	Hourly steps over one year	Hourly heat demand from smart meter data	HPs, RH, Gas Boilers, RH-B, HP-B, HP-RH	NaN
**MARKAL/ TIMES** [28]	Exploration	LP least-cost optimisation	National, regional	5-year steps until 2050 with 16 time slices	Daily heat demand profiles	Solar Thermal, HPs, RH, DHN, μ-CHPs	NaN
**CGEN** [29]	Investment	MINLP least-cost optimisation	Regional	5-year steps until 2050	Average annual and peak heat & electricity demand	NaN	NaN

of the GB. The authors concluded that even though the total gas demand for the low-pressure network is likely to decrease the peak-load demand in future scenarios was found to be increasing posing economic implications as far as maintenance and utilisation of the network were concerned. Love et al. using operational data of heat pumps in 696 domestic sites from the Renewable Heat Premium Payment scheme, investigated the impact on peak demand and ramp rates for the electricity system [34]. Scenarios with national heat pumps uptake up to 20% were examined and the authors concluded that: (i) maximum ramp rate is likely to increase by 0.3 (GW/half hour) compared to its current value, (ii) shift of the peak demand from the evening hours (17:00-18:00) to early morning (06:00-08:00) and (iii) increase of the peak demand by 7.5 GW. Dodds [28] using the UK MARKAL model investigated future scenarios for the residential heat sector under the 80% decarbonisation target by 2050. In his study, gas boilers remained predominant until 2030 providing around 90% of the heat demand while by 2050 around 60% of heat demand was provided by HPs. A more refined disaggregation of the domestic sector in the UK MARKAL model was employed in order to study changes in the fuel consumption and technology selection for heating. The results indicated that the disaggregation (36 house categories), does not change the overall fuel consumption however different policy insights can be generated due to selection of technologies in different house types.

With heat decarbonisation being a key priority area, the government and its independent bodies have published a number of reports, a timeline of which can be envisaged in Fig.4.

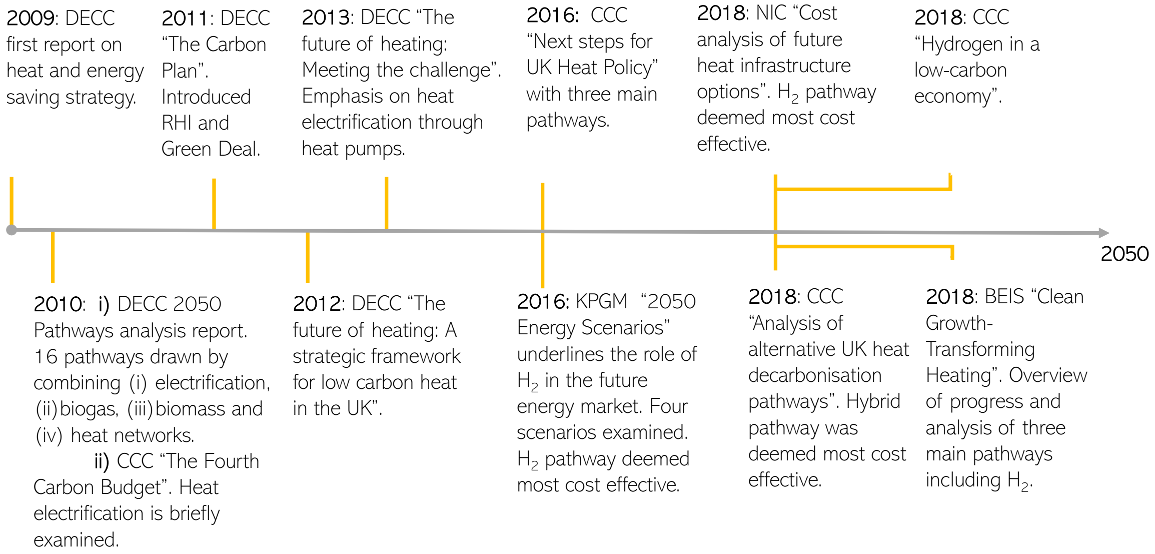

Figure 4: Indicative timeline of key policy and technical reports on heat decarbonisation for the UK.

In a report for the National Infrastructure Commission by Element Energy and E4tech, the cost of future decarbonisation pathways was examined [35]. Among the key findings was that regardless of the pathway, the heating cost is increased compared to the current heat infrastructure system with the annual average cost increase per household to be £100-300 in 2050. Deep electrification was reported to lead to additional peak demand of at least 45GW and the least-cost option was found to be the hydrogen pathway which however suffers from uncertainty

in terms of safety and technology costs. A hybrid gas-electricity infrastructure was found to be the second least-cost pathway while heat electrification with heat pumps was the most costly with the cumulative cost until 2050 reaching £270bn. The role of biomass gasification and biogas injection to the gas grid were also found to be low-regret options in some of the scenarios.

The cost of three heat decarbonisation pathways was also investigated by Imperial College London in their report for the Committee on Climate Change [36]. A variety of decarbonisation scenarios was considered with emission targets by 2050 ranging from 30-0 Mt/year. The hybrid gas-electric pathway was found to be the least-cost option under all scenarios while the hydrogen pathway was the most costly and most sensitive to variations in emission targets.

In contrast to the aforementioned works, a report published by the Energy Networks Association found that the decarbonisation pathway of hydrogen is the one resulting in the least capital and operational costs under the 2050 targets [37]. In this work, KPMG employed a top-down approach to evaluate four alternative scenarios including full electrification, two alternative hybrid pathways and the switch to hydrogen. Heat electrification was reported as the most expensive pathway with almost double the cost incurred by the hydrogen pathway. For the heat electrification scenario, the increased costs were attributed to the investments for nuclear and renewables energy sources as well as the reinforcement of the transmission network.

Supplementary note 2: Heat demand analysis

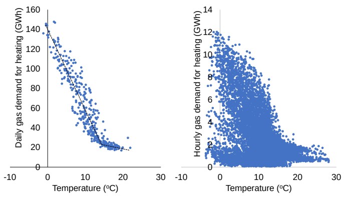

(a) East Anglia (EA) - 2015.

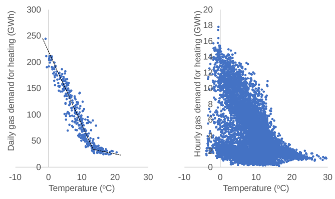

(b) East Midlands (EM) - 2015.

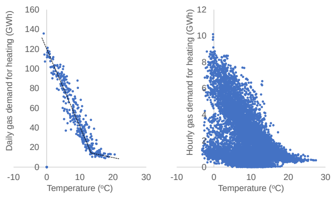

(c) North East (NE) - 2015.

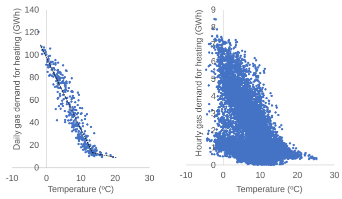

(d) North (NO) - 2015.

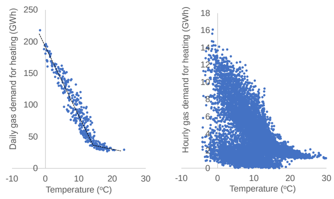

(e) North Thames (NT) - 2015.

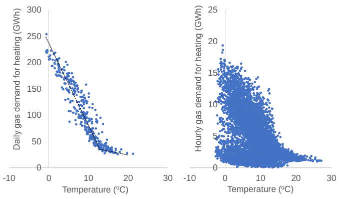

(f) North West (NW) - 2015.

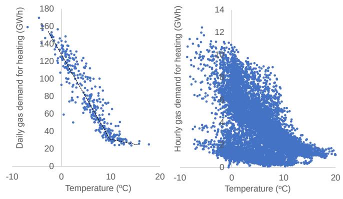

(g) Scotland (SC) - 2015.

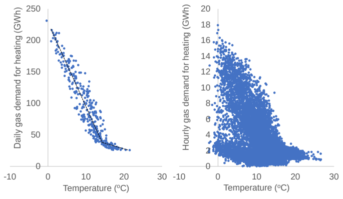

(h) South East (SE) - 2015.

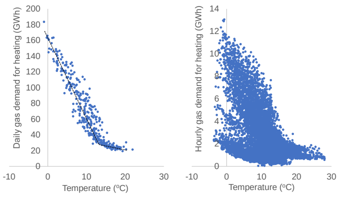

(i) South (SO) - 2015.

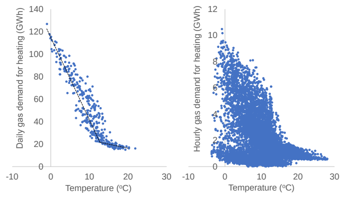

(j) South West (SW) - 2015.

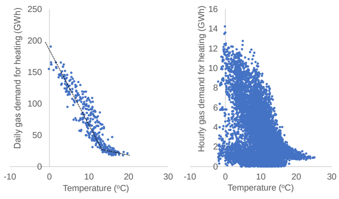

(k) West Midlands (WM) - 2015.

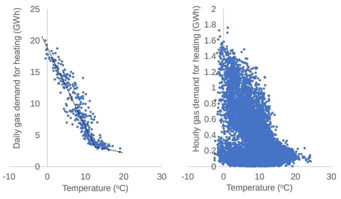

(l) Wales North (WN) - 2015.

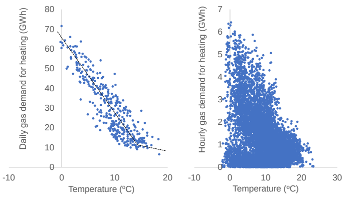

(m) Wales South (WS) - 2015.

Figure 5: Temperature dependence on daily (right graph) and hourly (left graph) domestic heat-related gas demand. Despite the temperature being one of the key factors that determine heat demand, the relationship on finer timescales (hourly) seems to be highly nonlinear. On the other hand, when the same data set is aggregated on a daily level the dependence between heat demand and temperature can be explained clearly via segmented linear regression.

Fig. 5 indicates the nonlinear nature of the correlation between heat-related gas demand and ambient temperature. While on a hourly level, the relationship between the two can be accurately

modelled following a piecewise linear regression, when examining the same data on a finer half-hourly scale, a nonlinear trend is observed which previous studies in Europe have also identified [38]. This gives further credence to the validity of the derived heat demand profiles as similar piecewise linear trends have been identified in past works on a daily scale [27, 36] and also highlights the importance of using actual historical demand data as they capture trends that the latter are unable to. In Fig. 6, a heat map that indicates the timings of regional peak demand during the so-called "Beast from the East" in GB during the period 28th of February - 1st of March 2018 is shown. While the GB-wide peak gas demand was on the 1st of March at 6pm from a regional perspective the peak gas consumption varied with regions like NT and SC having their peak demand on the 28th of February between 6-7pm.

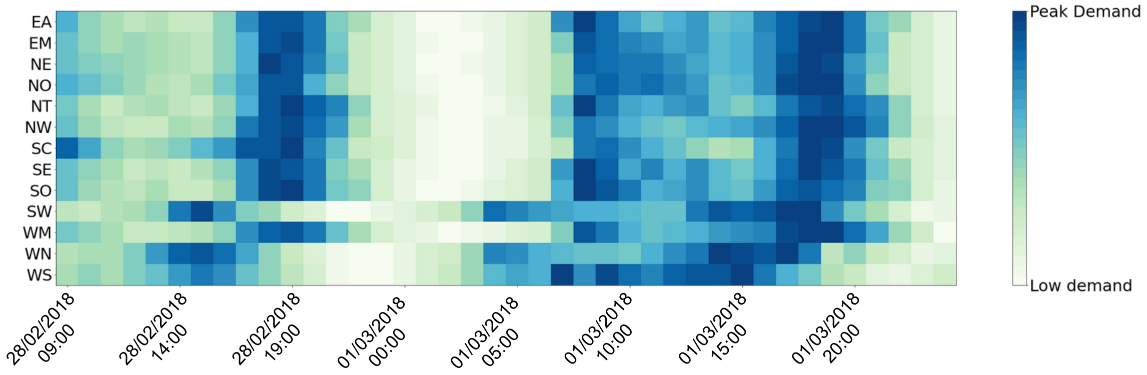

Figure 6: Heat map of regional heat demand during the period 28th of February - 1st of March 2018.

Finally, in Table 2 we provide the annual per household regional heat demand.

Table 2: Annual regional heat demand per household in GB (MWh) across the different LDZ regions

LDZ	Annual Heat Demand per household (MWh)
EA	12.35
EM	13.23
NE	12.51
NT	10.88
NO	11.35
NW	10.07
SC	10.30
SE	17.31
SO	9.79
SW	10.70
WM	10.26
WN	12.06
WS	11.38

Supplementary note 3: OPHELIA model overview

OPHELIA is formulated as a multiperiod spatially explicit mixed integer linear program which seeks to optimise simultaneously strategic and operational decisions by explicitly considering the electricity and heat sectors in Great Britain. The key assumptions of the model can be summarised as follows:

- Deterministic operational, cost and demand data;
- Centralised least-cost decision making;
- Electricity and gas demand are inelastic. Prices for interconnection are provided exogenously and future price projections are based on the European Network of Transmission System Operators' Electricity ten-year network development plan [39];
- Gas demand data are used as a proxy for the proportion of gas consumption that accounts for domestic heat demand [40];
- The model considers demand on a regional level for heat and electricity;
- Unmet electricity demand is penalised with a cost equal to the Value of Lost Load (VoLL) which is 6,000£/MWh [41];
- Unmet short term operating reserve requirements are penalised at 75£/MWh and 25 £/MWh for the cases of upward and downward reserve respectively while we assume an average cost of renewable generation curtailment of 47£/MWh [41];
- Regional distribution networks are not modelled and their respective losses are accounted by a factor proportional to the regional electricity demand [42];
- Transmission distances are calculated as the distance between the centroids of each region;
- Power plants' efficiencies are considered deterministic and heat rate curves for part-load are not considered;
- The regional allocation of Solar & Onshore Wind power plants is not constrained by land-availability restrictions;
- The allocation of nuclear & hydro power plants as well as new capacity of pumped hydro storage is restricted to regions where existing capacity is present;
- For generation capacity expansion we consider future projections of build rates over five-year planning steps based on [43].

Mathematical formulation

Objective function

OPHELIA's objective function is the minimisation of the total system's cost (TSC) comprising of: (i) total operating costs and (ii) total capital costs accounting for generation and transmission capacity expansion for the power sector and generation and storage capacity costs for the heat sector. Eq.(1) provides the summary of the terms comprising the TSC discounted by DFC_t.

$$min\, TSC = \sum_t DFC_t(TotCAPEX_t + TotOPEX_t) \tag{1}$$

Table 3: Modelling characteristics of the proposed approach.

Model Details	Description	Comments
**Modelled area**	Great Britain	
**Spatial resolution**	13 regions	GB is spatially disaggregated following the gas network LDZs.
**Temporal resolution**	Dual: 5-year strategic time steps with N_D representative days with hourly steps	Time-ordered statistical clustering of 24 hour intervals into representative days
**Electricity demand**	Non-heat and heat-driven demand	The heat-driven demand is optimised by the model
**Heat demand**	Hourly demand profiles by region	It is assumed that heat demand is derived by the corresponding gas consumption.

$$\begin{aligned} TotCAPEX_t = &\sum_{g,j \in J_{th}} C_j^{fix} Cap_j^{Unit} CUnits_{g,j,t} + \sum_{g,j \in J_{rew} \cap J_{es}} C_j^{fix} Cap_{g,j,t}^{New} \\ &+ \sum_{g,j \in JR_h \cap J_{h_s}} C_j^{fix} hcu_j CUnits_{g,j,r,t}^{heat} + \sum_{g,g' \in NG_{g,g'}} C_{tr}^{fix} LDD_{g,g'} TRI_{g,g',t}/2 \quad \forall t \end{aligned} \tag{2}$$

Eq. (2) summarises the total capital expenditure that accounts for investments for: (i) new power generation capacity, (ii) new grid-level storage capacity, (iii) new heat end-use technologies and (iv) new transmission grid capacity between regions.

$$\begin{aligned} TotOPEX_t = &\sum_{c,h} WD_c \Big(\sum_{g,j \in J_{th} \cap J_{rew}} C_j^{var} P_{g,j,c,h,t} + \sum_{j,f \in Jf_{j,f}} C_{f,t}^{fuel} V_{g,j,f,c,h,t}^{elec} \\ &+ \sum_{g,j \in J_{es}} C_j^{var} DC_{g,j,c,h,t} + \sum_{g,j \in J_{th}} (C_j^{start} Cap_j^{Unit} v_{g,j,c,h,t} + C_j^{shut} Cap_j^{Unit} w_{g,j,c,h,t}) \\ &+ \sum_{g,j \in J_{th}} C_j^{OM} Cap_j^{Unit} NUnits_{g,j,t} + \sum_{g,j \in J_{rew} \cap J_{es}} C_j^{OM} Cap_{g,j,t} \\ &+ \sum_{i,g \in IG,c,h,t} C_{i,g,c,h,t}^{INCX} PIM_{i,g,c,h,t} + C_t^{CO_2} (CO_{2t}^{elec} + CO_{2t}^{heat} - CO_2^{elec_{neg}}) \\ &+ C^{VoLL} \sum_g LShed_{g,c,h,t} + C^{CRTL} \sum_g LCurtail_{g,c,h,t} \\ &+ C^{STORdn} l_{c,h,t}^{OPRES_{down}} + C^{STORup} l_{c,h,t}^{OPRES_{up}} + \\ &\sum_{g,j,r \in JR_h \cap J_{h_s}} C_j^{OM} hcu_j NUnits_{g,j,r,t}^{heat} + \sum_{f \in Jf_{j_h,f}} C_{f,t}^{fuel} V_{g,j,f,c,h,t}^{heat} \Big) \quad \forall t \end{aligned} \tag{3}$$

Eq. (3) represents the total operating costs of the power and heat system and accounts for: variable costs for generation and storage (C_j^{var}) (which comprise of marginal OPEX and cost of CO_2 transport and storage where applicable); fuel consumption costs (C^{fuel}); fixed O&M

costs (C_j^{OM}) for power and heat generation and storage; cost of interconnection ($C_{i,g,c,h,t}^{INCX}$), which depending on the flow of electricity may reflect revenue or expenditure, cost of net CO_2 emissions from both sectors ($C_t^{CO_2}$) and finally penalties for shedding upward (C^{STORup}) or downward (C^{STORdn}) short term operating reserve, electricity demand (C^{VoLL}) or curtailing renewable generation (C^{CRTL}).

Heat supply chain

In this section the key constraints used to model the heat demand and generation components of the model are detailed. We assume that 23% of the total heat demand represents water heating load and the remaining for space heating in line with recent studies [26].

Heat balance

Eq.(4) represents the regional heat balance. In each region (g), on-gas or off-gas grid regional area (r), cluster (c), time-slice (h) and time-step (t) the heat demand and storage should be satisfied by the generation from different end-use technologies and the heat discharged.

$$HDem_{g,r,c,h,t} = \sum_{j \in JR_h} Q_{g,r,j,c,h,t}^{heat} + Q_{g,r,c,h,t}^{dheat} - Q_{g,r,c,h,t}^{sheat} \quad \forall g, r, c, h, t \tag{4}$$

The set JR_h is employed to allow for heat-end use technologies depending on whether the referred regional demand is connected to the gas grid. That is, off-gas grid regional demand is allowed to be served by heat electrification technologies only while on-grid demand can be satisfied by gas boilers or ASHPs, subject to emissions constraints.

Heat related power demand calculation

Eq.(5) calculates the resulting electricity demand due to heat electrification.

$$P_{g,r,c,h,t}^{heat} = \sum_{j \in \{ASHPs\}} \frac{Q_{g,r,j,c,h,t}^{heat}}{COP_{g,c,h,t}} \quad \forall g, r, c, h, t \tag{5}$$

The main modelling approaches for simulating the energy consumption and heat generation of ASHPs is through their coefficient of performance (COP). Despite COP being strongly dependent on the temperature of the energy source and the target temperature and thus time-varying, in some research works for the sake of simplicity it has been assumed as constant. Considering the COP of HPs as constant can lead to underestimation of the power required to meet the related heat demand and to this end a more elaborate calculation of the COP is pursued. Heinen et al. [22] proposed to calculate the COP of HPs as a linear function of the ambient temperature while piecewise linear functions have also been proposed in the literature [44, 45]. We should note, that for the case of centralised systems, such as DHN, the COP is assumed to be constant given that large-scale HPs are typically sourced from temperature-stable energy sources. Bach et al. [46] argued that the same assumption can be valid for the case of water-sourced heat pumps since the ambient temperature of the HP does not vary significantly throughout the year. To

allow for time-varying and regional COPs we employ eq.(6) which has been reported to preserve good accuracy over the performance range [45] whilst still being a linear function of the ambient temperature ($T^a_{g,c,h,t}$).

$$COP_{g,c,h,t} = 0.0541 T^a_{g,c,h,t} + 2.6674 \quad \forall g, c, h, t \tag{6}$$

It is worth noting that despite the input heat demand data were based on historic gas demand profiles, the hourly ASHPs electricity consumption profiles are computed and optimised endogenously by the model.

Heat technologies capacity constraints

Eq.(7) imposes that the heat generated by a specific end-use technology at any time should not exceed the existing installed capacity, while eq. (8) imposes that the number of installed units should be equal to the number of properties in each region (nH_g).

$$Q^{heat}_{g,j,r,c,h} \leq hcu_{j,g} NUnits^{heat}_{g,j,r,t} \quad \forall g, r, j \in JR_h, c, h, t \tag{7}$$

$$\sum_{j \in JR_h} NUnits^{heat}_{g,j,r,t} \leq nH_{g,r} \quad \forall g, j, r, t \tag{8}$$

The overall balance on heating technologies is given by eq. (9).

$$NUnits^{heat}_{g,j,r,t} = NUnits^{heat}_{g,j,r,t-1} + CUnits^{heat}_{g,j,r,t} - DUnits^{heat}_{g,j,r,t} - CUnits^{heat}_{g,j,r,t-tp_j} \quad \forall g, r, j \in J_h, t \tag{9}$$

Thermal energy storage constraints

We consider generic hot water tanks as thermal energy storage (TES). Eq. (10) is the heat storage balance where we consider a 5% self-discharge [47] for every hour. Eqs. (11)-(13) represent the allowable upper bounds on heat storage level ($S^{heat}_{g,j,r,c,h,t}$), charging ($Q^{sheat}_{g,j,r,c,h,t}$) and discharging ($Q^{dheat}_{g,j,r,c,h,t}$) amounts and related data are given in Table 9.

$$S^{heat}_{g,j,r,c,h,t} = S^{heat}_{g,j,r,c,h-1,t}(1 - \eta^{heat}_{storage}) - Q^{dheat}_{g,j,r,c,h,t} + Q^{sheat}_{g,j,r,c,h,t} \quad \forall g, j \in J_{hs}, r, c, h, t \tag{10}$$

$$S^{heat}_{g,j,r,c,h,t} \leq hcu_j Tes_j NUnits^{heat}_{g,j,r,t} \quad \forall g, j \in J_{hs}, r, c, h, t \tag{11}$$

$$Q^{dheat}_{g,j,r,c,h,t} \leq hcu_j NUnits^{heat}_{g,j,r,t} \quad \forall g, j \in J_{hs}, r, c, h, t \tag{12}$$

$$Q^{sheat}_{g,j,r,c,h,t} \leq hcu_j NUnits^{heat}_{g,j,r,t} \quad \forall g, j \in J_{hs}, r, c, h, t \tag{13}$$

$$NUnits^{heat}_{g,j,r,t} \leq \sum_{j \in J_{h_e}} NUnits^{heat}_{g,j,r,t} \quad \forall g, j \in J_{h_s}, t \tag{14}$$

$$S^{heat}_{g,j,r,c,h,t} = 0 \quad \forall g, j \in J_{h_s}, c, h = H, t \tag{15}$$

We only allow for deployment of TES in properties that have installed ASHPs and this is achieved by eq. (14) while in order to avoid energy cross-overs between representative days, eq. (15) imposes that the TES level empties at the end of each day.

Heating fuel consumption

The demand of heating fuel, i.e. natural gas, in the model is considered as endogenous. More specifically, demand of natural gas is related to the consumption by gas condensing boilers as shown by eq.(16).

$$V^{heat}_{g,j,r,f,c,h} = \frac{Q^{heat}_{g,r,c,h,t}}{\eta^{tech}_{g,j,c,h,t}} \quad \forall g, j \in J_h \wedge JF_{j,f}, f, c, h, r =' On - grid' \tag{16}$$

Power supply chain

Spatially explicit power balance

Eq.(17) presents the overall energy balance of the energy system on a temporal (c,h,t) and spatial granularity (g,g'). More specifically, the left-hand side of the equation refers to energy sources including: power produced ($P_{g,j,c,h,t}$), energy storage discharge ($DC_{g,j,c,h,t}$), power transmitted among neighbouring regions ($TR_{g',g,c,h,t}$) and for the regions who are interconnected the related power imports or exports ($PIM_{i,g,c,h,t}$). The right-hand side represents energy sinks for the system including: energy demand ($D_{g,c,h,t}$), energy storage charge ($CH_{g,j,c,h,t}$), transmission to neighbouring regions ($TR_{g,g',c,h,t}$), renewables' curtailment ($LCurtail_{g,c,h,t}$), power shedding ($LShed_{g,c,h,t}$).

$$\begin{aligned}
&\sum_{j \in J_e} P_{g,j,c,h,t}(1 - PL_j) + \sum_{j \in J_{es}} DC_{g,j,c,h,t} + \sum_{g' \in NG_{g,g'}} TR_{g',g,c,h,t}(1 - TL_{g',g} LDD_{g',g}) \\
&+ \sum_{i \in IG_{i,g}} PIM_{i,g,c,h,t}(1 - InterLoss_{i,g}) = D_{g,c,h,t}(1 + DL) - LShed_{g,c,h,t} + LCurtail_{g,c,h,t} \\
&\qquad + \sum_{g' \in NG_{g,g'}} TR_{g,g',c,h,t} + \sum_{j \in J_{es}} CH_{g,j,c,h,t} \quad \forall g, c, h, t
\end{aligned} \tag{17}$$

Notice that the electricity demand ($D_{g,c,h,t}$) further comprises of non-heat demand ($P^{elec}_{g,c,h,t}$) and demand resulting from electrification of heat ($P^{heat}_{g,r,c,h,t}$) as shown by eq.(18).

$$D_{g,c,h,t} = \sum_r P^{heat}_{g,r,c,h,t} + P^{elec}_{g,c,h,t} \quad \forall g, c, h, t \tag{18}$$

while the resulting system-wide peak demand ($PeakD_t$) for each planning time step is computed through eq. (19).

$$PeakD_t \geq \sum_g D_{g,c,h,t} \quad \forall t \tag{19}$$

Finally the fuel consumption for power generation of thermal power plants is computed by eq.(20).

$$V^{elec}_{g,j,f,c,h,t} = \frac{P_{g,j,c,h,t}}{\eta^{tech}_{g,j,c,h,t}} \quad \forall g, j \in J_{th}, f \in Jf_{j,f}, c, h, t \tag{20}$$

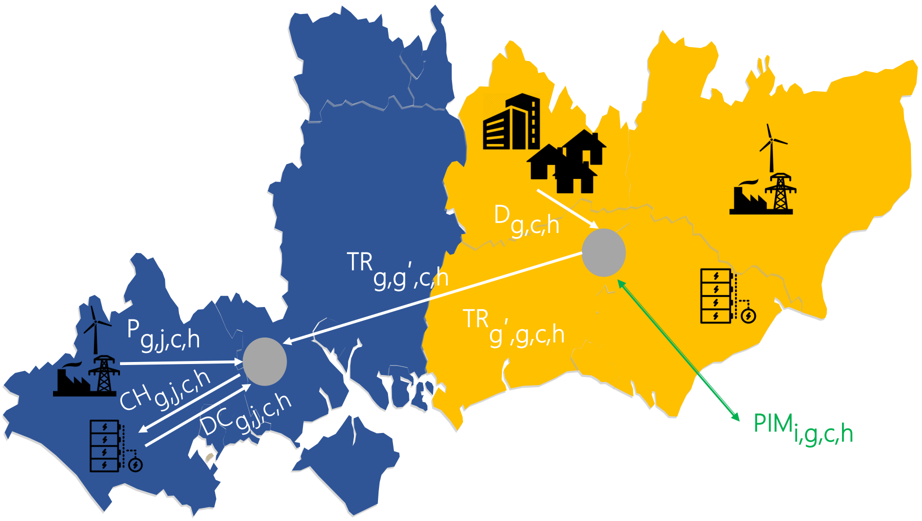

Figure 7: Spatially explicit energy balance for regions g and g′ and a fixed year t.

Transmission and interconnection

In the case that a region cannot meet its power demand from its regional power generation, electricity can be transmitted between regions. The amount of electricity transmitted between regions is limited by the capacity of the transmission network ($TRC_{g,g'}$) as shown by eq. (21).

$$TR_{g,g',c,h,t} \leq TRC_{g,g',t} \quad \forall (g,g') \in NG_{g,g'}, c, h, t \tag{21}$$

$$TRC_{g,g',t} = TRC_{g,g',t-1} + TRI_{g,g',t} \quad \forall (g,g') \in NG_{g,g'}, t \tag{22}$$

$$TRC_{g,g',t} = TRC_{g',g,t} \quad \forall (g,g') \in NG_{g,g'}, t \tag{23}$$

$$TRI_{g,g',t} \leq TRI^{up} \quad \forall (g,g') \in NG_{g,g'}, t \tag{24}$$

Overall, eqs. (21)-(24) model transmission corridors between regions with existing transmission capacity as well as transmission capacity expansion balance and investment limits (TRI^{up}). For the case of interconnection, interconnection capacities are considered deterministic and as model inputs based on the data given in Table 10. Eqs. (25)-(26), impose upper and lower limits on the bi-directional flow of electricity ($PIM_{i,g,c,h,t}$).

$$PIM_{i,g,c,h,t} \leq ICap_{i,g,t} \quad \forall (i,g) \in IG_{i,g}, c, h, t \tag{25}$$

$$PIM_{i,g,c,h,t} \geq -ICap_{i,g,t} \quad \forall (i,g) \in IG_{i,g}, c, h, t \tag{26}$$

Unit commitment

On the operational level of the model, decisions are modelled following the unit commitment (UC) formulation [48–50]. In order to rigorously model normal operation, start-ups and shut-downs of the generation plants the variables $u_{g,j,h}$, $v_{g,j,h}$, $w_{g,j,h}$ are accordingly employed.

$$u_{g,j,c,h,t} = u_{g,j,c,h-1,t} + v_{g,j,c,h,t} - w_{g,j,c,h,t} \quad \forall g, j \in J_{th}, c, h, t \tag{27}$$

$$\sum_{h'=h-UT_j}^{h} v_{g,j,c,h',t} \leq u_{g,j,c,h,t} \quad \forall g, j \in J_{th}, c, h \in [UT_j + 1, H], t \tag{28}$$

$$u_{g,j,c,h,t} \leq NUnits_{g,j,t} \quad \forall g, j \in J_{th}, c, h, t \tag{29}$$

$$\sum_{h'=h-DTj}^{h} w_{g,j,c,h',t} \leq NUnits_{g,j,t} - u_{g,j,c,h,t} \quad \forall g, j \in J_{th}, c, h \in [DT_j + 1, H], t \tag{30}$$

Eqs.(27)-(30) are employed to model the operational status of the generators clusters as well as the related start-up and shut-down events. Notice that eqs.(28)-(30) mathematically represent the polytope of the minimum up/down constraints and constitute the most computationally efficient formulation [51, 52].

Power generation limits

Each thermal generation plant has specified minimum (P_j^{min}) and maximum (P_j^{max}) stable thermal generation limits to model this, we employ eqs.(31)-(32).

$$P_j^{min} Cap_j^{Unit} u_{g,j,c,h,t} \leq P_{g,j,c,h,t} + r^{down}_{g,j,c,h,t} \quad \forall g, j \in J_{th}, c, h, t \tag{31}$$

$$P_{g,j,c,h,t} + r^{up}_{g,j,c,h,t} \leq P_j^{max} Cap_j^{Unit} u_{g,j,c,h,t} \quad \forall g, j \in J_{th}, c, h, t \tag{32}$$

Notice that in case that no generator is committed, i.e. $u_{g,j,h} = 0$, then the power generated is forced to be zero as well. The variables $r^{down}_{g,j,c,h}$ and $r^{up}_{g,j,c,h}$ refer to the participation in downward and upward operating reserve market respectively.

On the other hand, intermittent renewables energy sources ($j \in J_{rew}$) most of the times are not dispatchable immediately when required and their power production in depended weather factors such as solar irradiation and wind speed. To account for this issue eq.(33) is used and the parameter $AV_{g,j,h}$ defines the availability of the renewable source as a fraction of the installed capacity.

$$P_{g,j,c,h,t} = AV_{g,j,c,h,t} Cap_{g,j,t} \quad \forall g, j \in J_{rew}, c, h, t \tag{33}$$

$$LCurtail_{g,c,h,t} \leq \sum_{j \in J_{rew}} P_{g,j,c,h,t} \quad \forall g, c, h, t \tag{34}$$

Eq.(34) secures that the amount of curtailed power at any time and region should be less or equal to the amount of power generated by renewable sources.

Thermal generation ramping limits

Operations of conventional thermal generation plants are restricted by their hourly ramp-up and ramp-down capabilities. To model this, eq. (35) is used for the case of ramp-up rates with consideration of different capabilities during the start-up (SU_j) and the committed phase (RU_j).

$$\begin{aligned} P_{g,j,c,h,t} - P_{g,j,c,h-1,t} \leq\ & SU_j Cap_j^{Unit} v_{g,j,c,h,t} \\ & + RU_j Cap_j^{Unit} (u_{g,j,c,h,t} - v_{g,j,c,h,t}) - r^{up}_{g,j,c,h,t} \quad \forall g, j \in J_{th}, c, h, t \end{aligned} \tag{35}$$

Equivalently, to model ramp-down restrictions of thermal generation plants eq.(36) is employed and similarly to above we model different capabilities during the shut-down (SD_j) and the committed phase (RD_j).

$$\begin{aligned} P_{g,j,c,h-1,t} - P_{g,j,c,h,t} \leq\ & max[SD_j, P_j^{min}] Cap_j^{Unit} w_{g,j,c,h,t} \\ & + RD_j Cap_{g,j} (u_{g,j,c,h,t} - v_{g,j,c,h,t}) - r^{down}_{g,j,c,h,t} \quad \forall g, j \in J_{th}, c, h, t \end{aligned} \tag{36}$$

Operating reserve modelling & requirements

In order to account for upward and downward operating reserve requirement eq.(37)-(39) are used.

$$\sum_{j \in J_{th}, g} (r^{up}_{g,j,c,h,t} + r^{up_{nonsync}}_{g,j,c,h,t}) + \sum_{j \in J_{es}, g} r^{up_{nonsync}}_{g,j,c,h,t} = OPRES^{up}_{c,h,t} - l^{OPRES_{up}}_{c,h,t} \quad \forall c, h, t \tag{37}$$

$$\sum_{j \in J_{th}, g} r^{down}_{g,j,c,h,t} = (OPRES^{down}_{c,h,t} - l^{OPRES_{down}}_{c,h,t}) \quad \forall c, h, t \tag{38}$$

$$r^{up_{nonsync}}_{g,j,c,h,t} \leq (NUnits_{g,j,t} - u_{g,j,c,h,t}) * max(P_j^{min}, SU_j) Cap_j^{Unit} \quad \forall g, j \in J_{th}, c, h, t \tag{39}$$

In eq.(37) the variable $OPRES^{up}_{c,h,t}$ represents the system-wide requirement for upward operating reserve while the decision variable $l^{OPRES_{up}}_{g,c,h,t}$ stands for the amount of load shedding for upward operating reserve. Similar quantities were introduced in eq.(38). We also account for non-synchronous participation ($r^{up_{nonsync}}_{g,j,c,h,t}$) in the reserve markets for thermal generators that are not committed, via eq. (39) and for grid-level storage units. The upward ($OPRES^{up}_{g,c,h,t}$) and

downward ($OPRES^{down}_{g,c,h,t}$) reserve requirements are computed endogenously by OPHELIA based on eqs. (40)-(41).

$$OPRES^{up}_{g,c,h,t} = D^{error}_{c,h} \sum_{g} D_{g,c,h,t} + Res^{Error}_{c,h} \sum_{g,j \in J_{res}} AV_{g,j,c,h,t} Cap_{g,j,t} + SCap^{N-1} \quad \forall c,h,t \quad (40)$$

$$OPRES^{down}_{g,c,h,t} = 0.5(D^{error}_{c,h} \sum_{g} D_{g,c,h,t} + Res^{Error}_{c,h} \sum_{g,j \in J_{res}} AV_{g,j,c,h,t} Cap_{g,j,t} + SCap^{N-1}) \quad \forall c,h,t \quad (41)$$

As shown by eqs. (40)-(41) the short term operating reserve is a function of the demand error and generation output error of renewable energy sources along with the N-1 security consideration ($SCap^{N-1}$). The downward operating reserve is parameterised as 50% of the upward reserve requirement.

Energy storage constraints

Energy storage is modelled using eqs.(42)-(45). Charging (eq. (42)) and discharging (eq. (43)) is restricted by the installed capacity. The total energy volume stored cannot exceed storage volume capacity (which is defined as its storage capacity times maximum output hours at that capacity, eq. (44)). Finally, eq. (45) makes sure that total energy discharging cannot exceed the energy volume that was stored before ($ST^{init}_{g,j,t}$) and total net charging during the modelling horizon.

$$CH_{g,j,c,h,t} \leq Cap_{g,j,t} \quad \forall g, j \in J_{es}, c, h, t \quad (42)$$

$$DC_{g,j,c,h,t} \leq Cap_{g,j,t} \quad \forall g, j \in J_{es}, c, h, t \quad (43)$$

$$\sum_{h'|h' \leq h} (\eta_j CH_{g,j,c,h',t} - DC_{g,j,c,h',t}) + ST^{init}_{g,j,t} \leq Cap_{g,j,t} ST^{out}_{j} \quad \forall g, j \in J_{es}, c, h, t \quad (44)$$

$$\sum_{h'|h' \leq h} (DC_{g,j,c,h',t} - \eta_j CH_{g,j,c,h',t} + r^{up_{nonsync}}_{g,j,c,h,t}) \leq ST^{init}_{g,j,t} \quad \forall g, j \in J_{es}, c, h, t \quad (45)$$

$$ST^{init}_{g,j,t} = 0.5 Cap_{g,j,t} \quad \forall g, j \in J_{es}, t \quad (46)$$

Capacity expansion constraints

We consider capacity expansion of power generation units over five-year time steps (t) along with the initially available generation fleet and their projected decommissioning timings at the end of their operating lifetime. In summary the constraints employed are given by eqs. (47)-(51).

$$NUnits_{g,j,t} = NUnits_{g,j,t-1} + CUnits_{g,j,t} - DUnits_{g,j,t} - CUnits_{g,j,t-tp_j} \quad \forall g, j \in J_{th}, t \tag{47}$$

$$Cap_{g,j,t} = Cap_{g,j,t-1} + Cap^{New}_{g,j,t} - DCap_{g,j,t} - Cap^{New}_{g,j,t-tp_j} \quad \forall g, j \in J_{rew} \cup J_{es}, t \tag{48}$$

$$\sum_{g,j \in J_{th}} DF_j Cap^{Unit}_j NUnits_{g,j,t} + \sum_{g,j \in (J_{rew} \cup J_{es})} DF_j Cap_{g,j,t} \geq (1 + RM) PeakD_t \quad \forall t \tag{49}$$

$$CUnits_{g,j,t} \leq BR_{j,t} \Delta t \quad \forall g, j \in J_{th}, t \tag{50}$$

$$Cap^{New}_{g,j,t} \leq BR_{j,t} \Delta t \quad \forall g, j \in J_{rew} \cup J_{es}, t \tag{51}$$

Eqs. (47)-(48) represent the capacity balances for thermal and renewables/energy storage units respectively. While for the case of thermal generation we consider integer number of identical units ($NUnits_{g,j,t}$) with fixed unit capacity (Cap^{Unit}_j) for the case of RES and energy storage we allow the capacity to be a continuous variable ($Cap_{g,j,t}$) owing to the flexibility in capacity sizes. Eq. (49) reflects the planning reserve requirement which indicates that the system's installed capacity, after considering for technology-specific de-rating factors [53], should be at least equal to the peak demand plus a reserve margin factor which in our case is taken to be 13%. Finally, eqs. (50)-(51) impose new capacity deployment restrictions based on build rates ($BR_{j,t}$) over the five-year time steps (Δt).

Carbon emissions constraint

We consider separate carbon emissions targets for the electricity and the heat sectors. For the power sector carbon emissions targets are set as carbon budgets as advised by the UK Committee on Climate Change [54]. For the heat sector we linearly extrapolate from 2018 to 2053 towards gross zero heat emissions as we do not allow for negative emissions to be allocated for the heat carbon budgets. Eqs. (52)-(53) are used to monitor the emissions from the heat sector while eqs. (54)-(56) are employed for the power sector.

$$CO_{2t}^{heat} \leq \overline{CO_2^{heat}}_t \quad \forall t \tag{52}$$

$$CO_{2t}^{heat} = \sum_{g,j \in J_h, f \in JF_{j,f}} \varepsilon_f \sum_{c,h} WD_c V_{g,j,f,r,c,h,t}^{heat} \quad \forall t \tag{53}$$

$$CO_{2t}^{elec} - CO_{2t}^{elec_{neg}} \leq \overline{CO_2^{elec}}_t \quad \forall t \tag{54}$$

$$CO_{2t}^{elec} = \sum_{g,j \in J_{th}, f \in JF_{j,f}} \varepsilon_f \sum_{c,h} WD_c (1 - CCS_j^{rem}) V_{g,j,f,c,h,t}^{elec} \quad \forall t \tag{55}$$

$$CO_{2t}^{elec_{neg}} = - \sum_{g,j \in J_{neg}, f \in JF_{j,f}} \varepsilon_f \sum_{c,h} WD_c CCS_j^{rem} V_{g,j,f,c,h,t}^{elec} \quad \forall t \tag{56}$$

Model implementation and solution procedure

The overall OPHELIA model is formulated as a mixed integer linear program (MILP) and is implemented in GAMS 31.2 and solved with CPLEX 12.1 on a computer cluster composed of 32 machines with a total of 384 GB RAM (24 threads). Solving the model in a monolithic fashion, is computationally prohibitive as the for perfect foresight and 12 representative days the model comprises of 3.8 million equations, 6 million constraints and 500,000 discrete variables. To this end, we employ a myopic rolling horizon procedure in which the we decompose the planning periods in bins of two adjacent periods and each time one bin is solved. Then all the design decisions are fixed and the planning horizon is shifted by two time steps and the procedure is repeated until the entire horizon is covered. Following this procedure the model is solved to optimality gap of 3% in 22-26 hours depending on the scenario and input data.

Supplementary note 4: OPHELIA summary of data

Electricity demand data

Electricity demand data for GB are publicly available on a half-hourly and nation-wide basis [55]. First, aggregate and average out the half-hourly data to hourly resolution. Then in order to disaggregate the data regionally we use the sub-national electricity consumption statistics [56] and compute the regional demand shares for its LDZ as shown in Table 4.

Transmission grid & interconnection assumptions and data

To model the transmission capability of the system across the different regions, the system technical data from the ten-year grid statement are used [57]. Due to the scope of the present to model simultaneously the UK electricity and gas system we aggregate the transmission line capacities into the 13 regions considered in the model. To do so, the line capacities of the National Grid's ten year statement technical data are employed. In the technical data the seasonal line capacities are detailed with the summer being around 80% of the winter's rating [57]. Aggregating all the line capacities that cross a local distribution zone's borders using the 2018 data and assuming that transmission is done between the centroids of the regions we calculate the linear

Table 4: Electricity demand allocation per region

Local Distribution Zone (LDZ)	Share (%)
EA	9.6
EM	7.5
NE	6.5
NO	5.0
NT	13.9
NW	12.1
SC	8.9
SE	2.90
SO	10.9
SW	8.6
WM	8.7
WN	1.5
WS	3.9

distance (LDD) and transmission capacities between LDZs ($TRC_{g,g',t}$) as shown in Table 5. Transmission losses are assumed to be 1% per 100km [58]. A graphical representation is given in Fig.8. UK security standards require the transmission system to endure failure of up to two circuits (N-2 security rule). Other models such as highRES [58] and ELMOD[59] impose a security margin between 20-25% on the nominal transmission capabilities to simulate a more realistic operation of the system. Nonetheless, the proposed model can be easily modified to account for such parametrisation. As far as interconnection prices of third countries are concerned, we use the future projected prices of the "Global Ambition" scenario from the Ten Year Network Development Plans of the European Network of Transmission System Operators for Electricity [39]. Finally, in Table 5 the techno-economic data of the transmission system are given.

Table 5: Cross-regional transmission data (Overground 75 km 400kV (AC)).

Transmission Characteristics	Value	Source
Marginal CAPEX (£/MVA/km)	247	[58]
Transmission losses (%/100km)	1	[58]
Lifetime (years)	40	[58]

Power generation technology data

OPHELIA considers existing power plant's capacities across the regions along with their decommissioning at the end of their lifetime based on the data available from BEIS [60]. A summary of the techno-economic characteristics for the thermal generation technologies is given in Table 6 while for renewable energy sources the relevant data are summarised in Table 7.

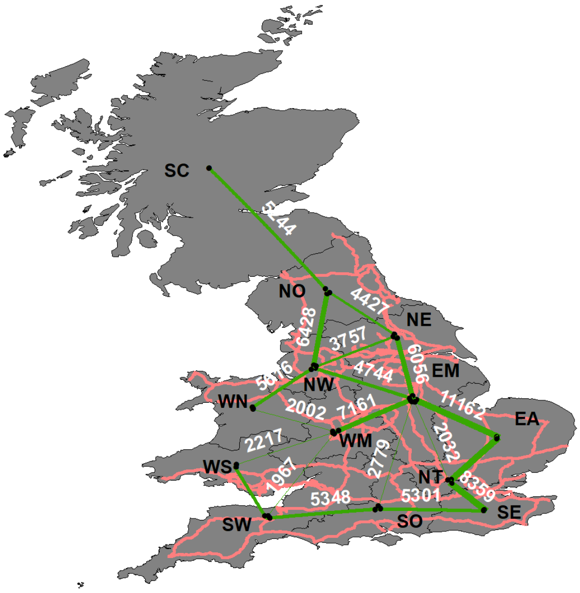

Figure 8: Initial cross-regional transmission capacities

Table 7: Techno-economic specifications of RES technologies.

	Onshore Wind	Offshore Wind	Solar PV	Source
Fixed O & M (£'000/MW-year)	23.5	28	6.7	[67]
Capital cost (£'000/MW)	1400	2100	750	[67]
Variable cost (£/MWh)	0	0	0	[67]
Lifetime (years)	25	30	30	[67]
De-rating factor (%)	0.25	0.36	0.14	[67]

Table 6: Techno-economic specifications of thermal generation technologies.

	Nuclear	Coal	CCGT	CCGTCCS	OCGT	Biomass	BECCS	Sources
Net efficiency (LHV) (%)	0.391	0.356	0.515	0.49	0.39	0.35	0.28	[61–63]
Min uptime (h)	12	8	2	4	1	4	8	[61–63]
Min downtime (h)	12	8	2	4	1	4	8	[61–63]
Min load (%)	0.5	0.35	0.35	0.5	0.3	0.4	0.5	[61, 62]
Max load (%)	0.88	0.8	0.87	0.8	0.94	0.8	0.8	[61–63]
Parasitic load (%)	0.0917	0.0513	0.0171	0.12	0.0171	0.0513	0.2	[61–63]
Committed Ramp up (%)	0.1	0.3	1	1	1	0.3	0.3	[61, 62]
Committed Ramp down (%)	0.1	0.3	1	1	1	0.3	0.3	[61, 62]
Start-up Ramp up (%)	0.3	0.3	1	1	1	0.3	0.3	[61, 62]
Shut-down Ramp down (%)	0.3	0.3	1	1	1	0.3	0.3	[61, 62]
Start-up cost (£/MW-start-up)	22.4	72.4	54.2	22.06	64.5	65.2	44.9	[61, 62, 64]
Fixed O & M (£'000/MW-year)	72.94	56.4	11.44	30.98	4.57	65.5	139.14	[61, 62, 65]
Capital cost (£'000/MW)	4435.5	1237	583.5	2100	750	3075	5885.5	[61, 62, 65]
CO_2 capture (%)	-	-	-	0.9	-	-	0.9	[61, 66]
Variable cost (£/MWh)	2.62	2.2	1.43	2.5	1.91	4	7.86	[61, 62, 65]

Table 8: Energy storage technologies characteristics. As a generic grid-level storage technology we consider NaS (sodium-sulfur) battery. For pumped hydro storage we consider the medium cost estimates from [61]. For domestic thermal energy storage we consider hot water tanks.

Storage Characteristics	Pumped storage	Grid-level storage	Thermal storage	Source
Marginal CAPEX (10^3£/MW)	1032	612	35	[47, 61, 68]
Variable OPEX (£/MWh)	10	2	8	[26, 47, 68]
Fixed O&M (£/kW-year)	11.2	3.6	incl. in Capex	[47, 61, 68]
Round-trip efficiency (%)	77	86	-	[20, 61, 68]
Power to Energy ratio	8	6	4	[26, 58, 61, 68]
Thermal losses per hour (%)	-	-	5	[22, 47]
Lifetime (years)	50	20	20	[26, 61, 68]

Table 9: Heat end-use technologies data.

Technology Characteristics	Gas boiler	ASHP	Source
Marginal CAPEX (10^3£/MW)	75	612	[36]
Fixed O&M (£/kW$_{th}$-year)	32	20	[22, 26]
Lifetime (years)	15	15	[22, 26]
Efficiency (%)	90	160-350	[22, 26]

Table 10: Interconnection data [69, 70].

Interconnection	Connected markets	LDZ connection	Capacity (GW)	Thermal losses (%)
IFA	France	SE	2	1.17
MOYLE	Irish SEM	SC	0.5	2.36
BritNed	Netherlands	SE	1	3.45
EWIC	Irish SEM	WN	0.5	4.68
NEMO	Belgium	SE	1	2.67
Eleclink	France	SE	1	2.08
IFA2	France	SO	0.5	4.68
NSS	Norway	NO	1.5	7.98
Greenlink	Irish SEM	WS	0.5	3.30
Fablink	France	SW	1.4	4.68
Vikinglink	Denmark	EM	1	6.90
Northconnect	Norway	SC	1.4	7.35

Supplementary note 5: Overview of regional results

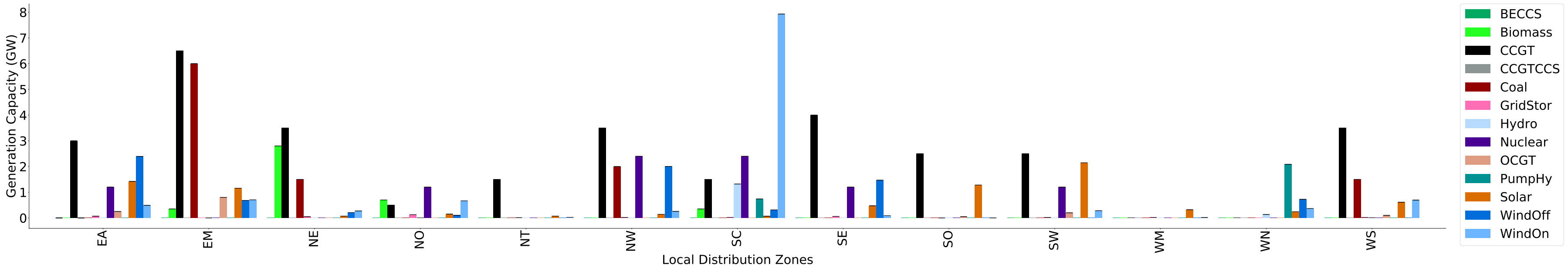

(a) Installed generation capacities across LDZs in 2018 (input data).

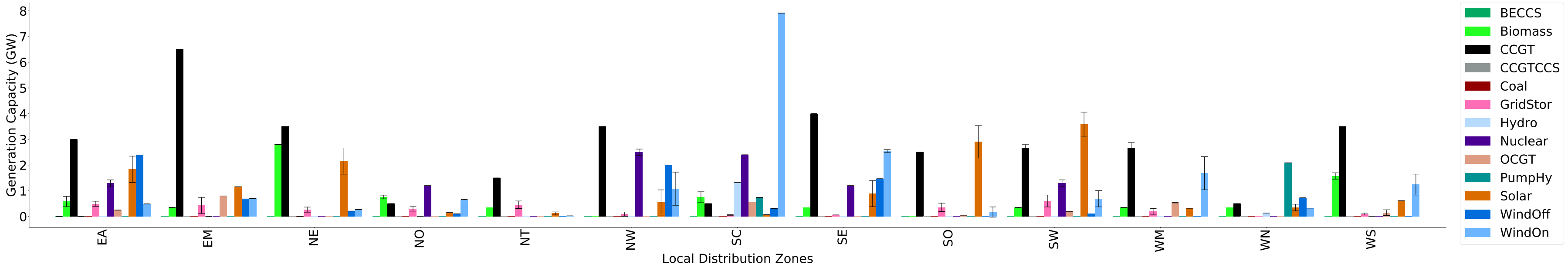

(b) Installed generation capacities across LDZs in 2023 (model output).

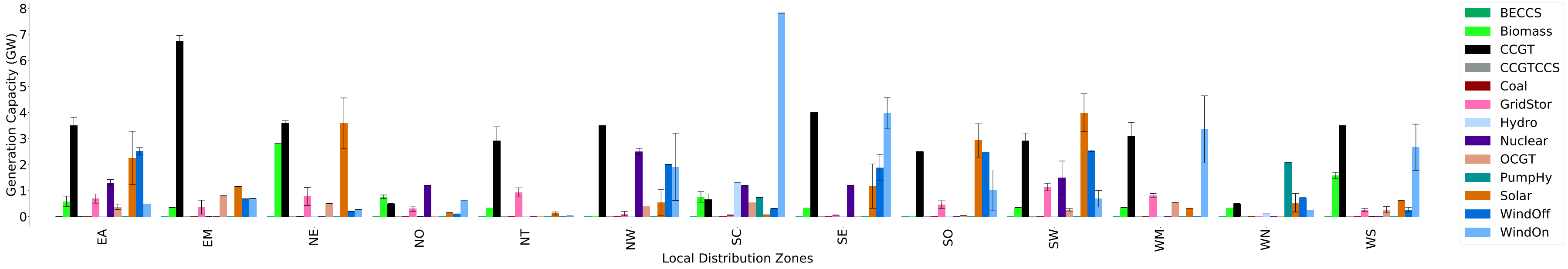

(c) Installed generation capacities across LDZs in 2028 (model output).

Figure 9: Optimised regional allocation of generation capacities for 2018-2053. The error bars of the individual bars indicate the variation resulting from running the optimisation model with different heat demand data from 2015-2018.

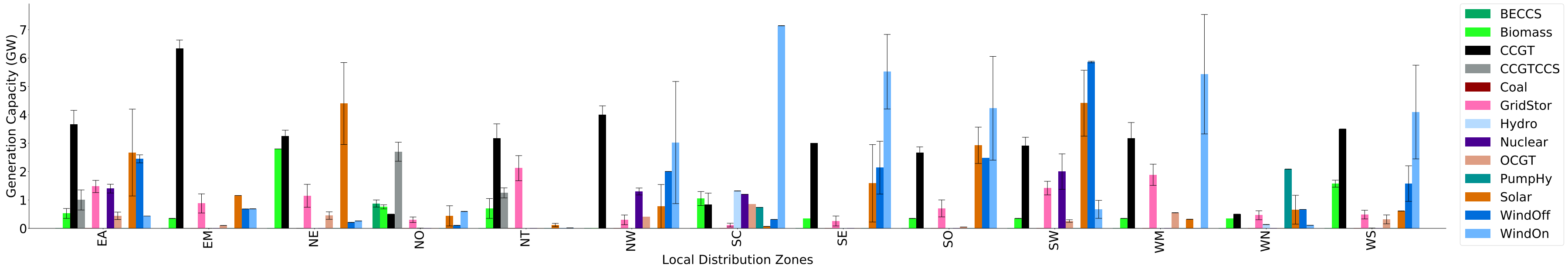

(a) Installed generation capacities across LDZs in 2033 (model output).

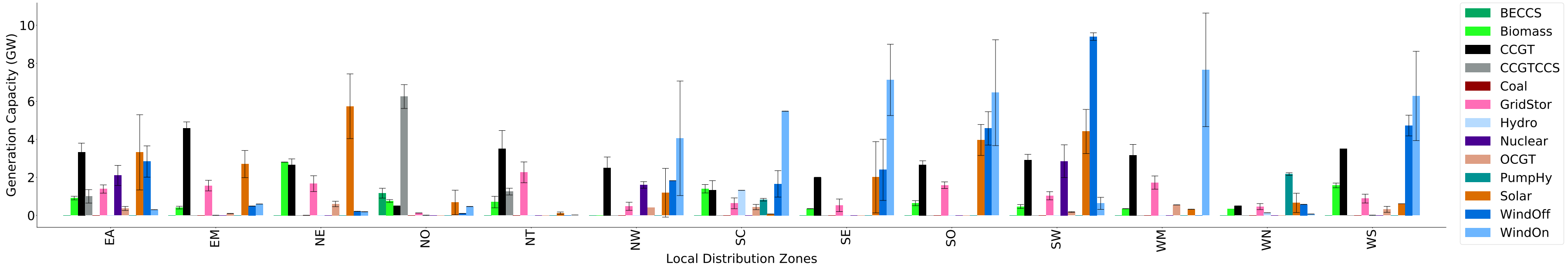

(b) Installed generation capacities across LDZs in 2038 (model output).

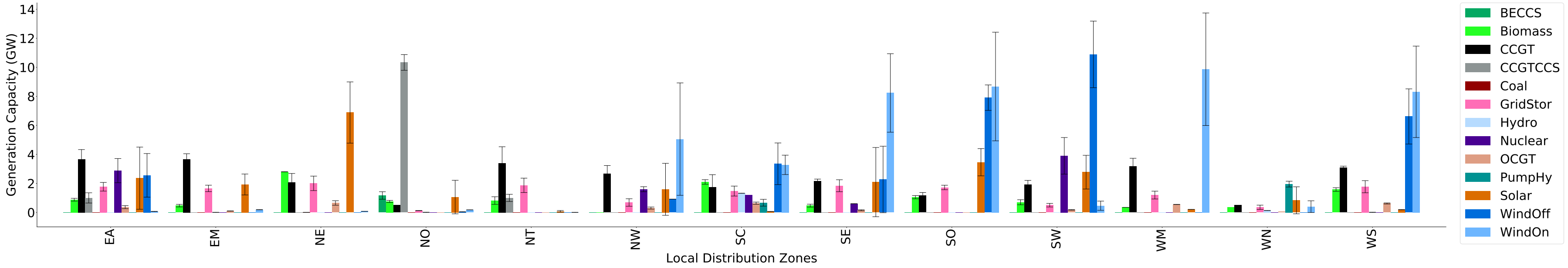

(c) Installed generation capacities across LDZs in 2043 (model output).

Figure 9: (continued) Optimised regional allocation of generation capacities for 2018-2053. The error bars of the individual bars indicate the variation resulting from running the optimisation model with different heat demand data from 2015-2018.

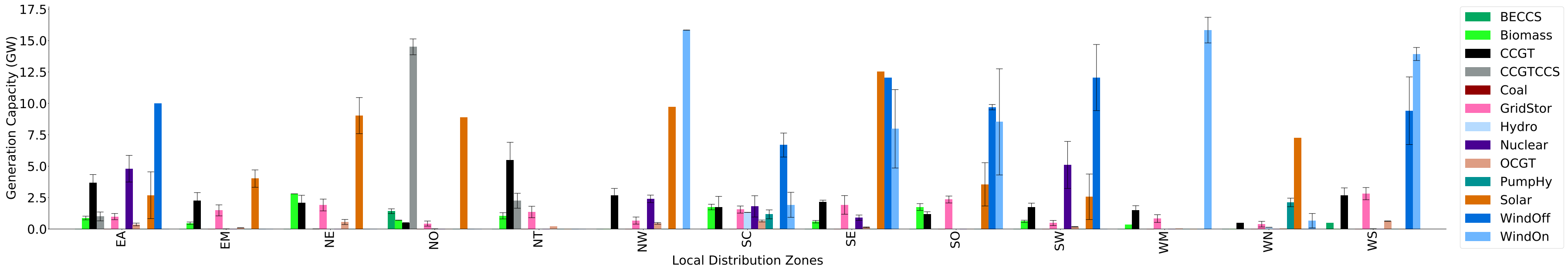

(d) Installed generation capacities across LDZs in 2048 (model output).

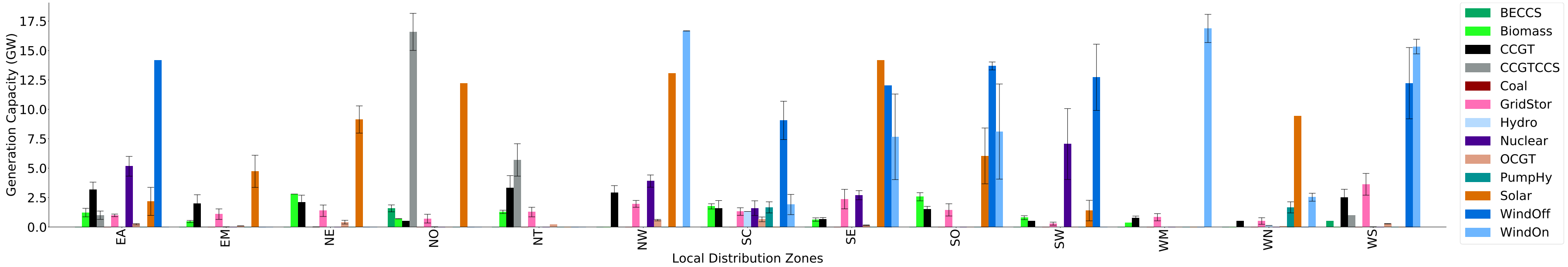

(e) Installed generation capacities across LDZs in 2053 (model output).

Figure 9: (continued) Optimised regional allocation of generation capacities for 2018-2053. The error bars of the individual bars indicate the variation resulting from running the optimisation model with different heat demand data from 2015-2018.

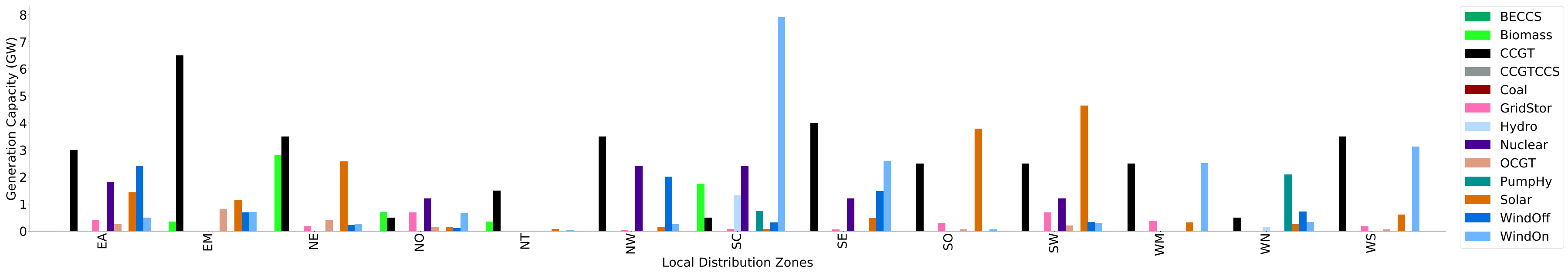

(f) Installed generation capacities across LDZs in 2023 (model output).

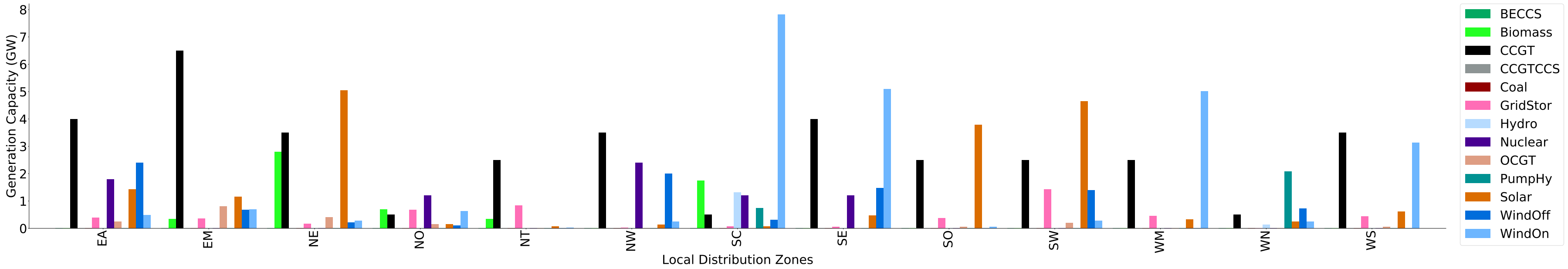

(g) Installed generation capacities across LDZs in 2028 (model output).

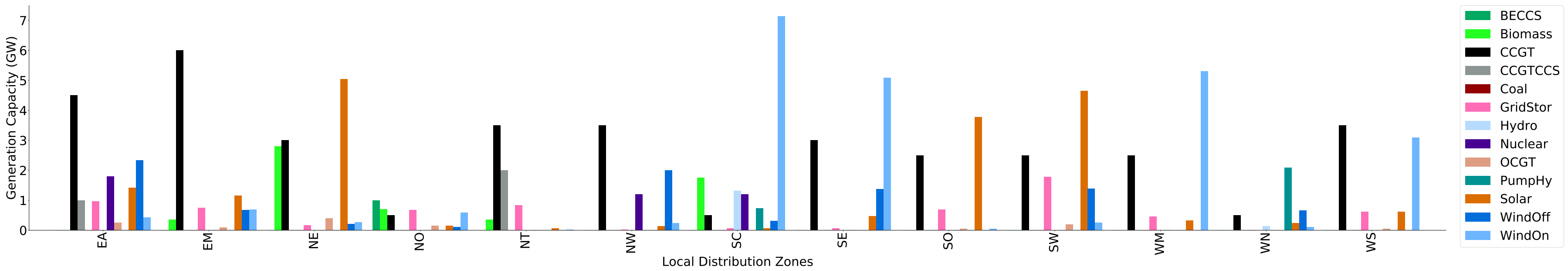

(h) Installed generation capacities across LDZs in 2033 (model output).

Figure 10: Optimised regional allocation of generation capacities for 2018-2053 for power sector only decarbonisation.

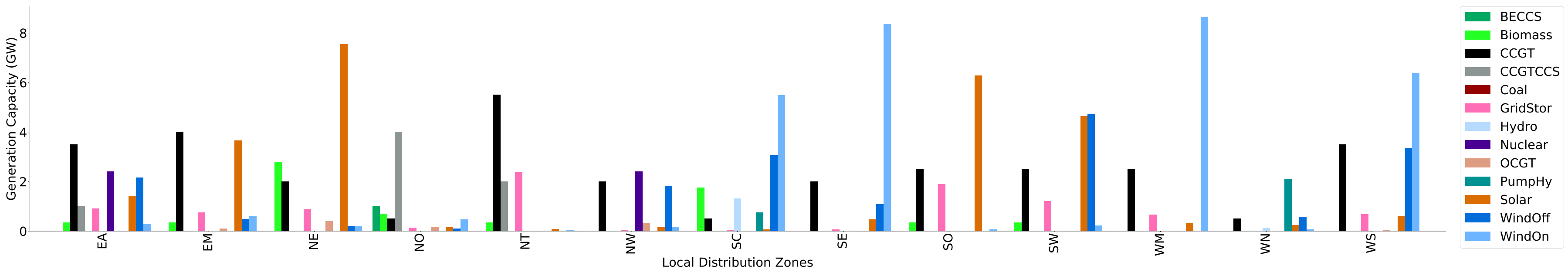

(a) Installed generation capacities across LDZs in 2038 (model output).

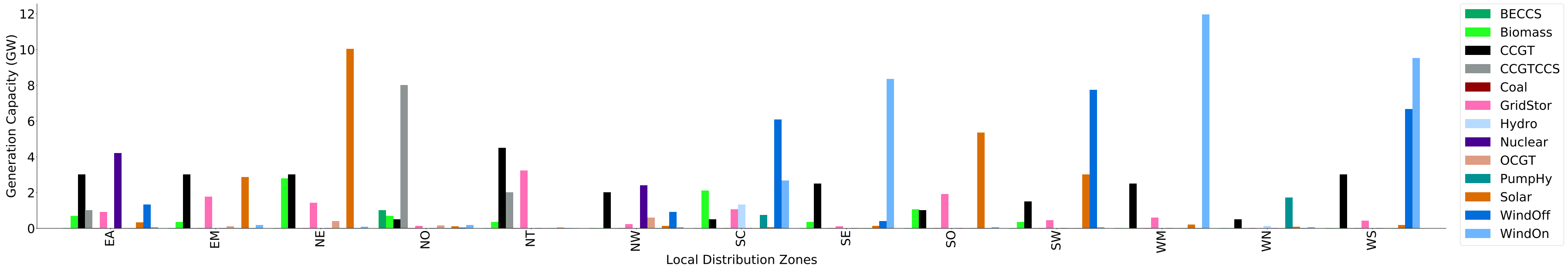

(b) Installed generation capacities across LDZs in 2043 (model output).

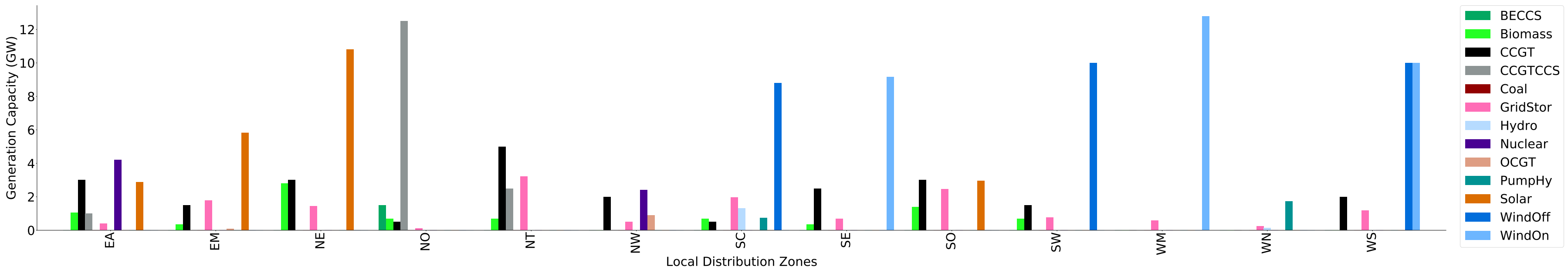

(c) Installed generation capacities across LDZs in 2048 (model output).

Figure 10: (continued) Optimised regional allocation of generation capacities for 2018-2053 for power sector only decarbonisation.

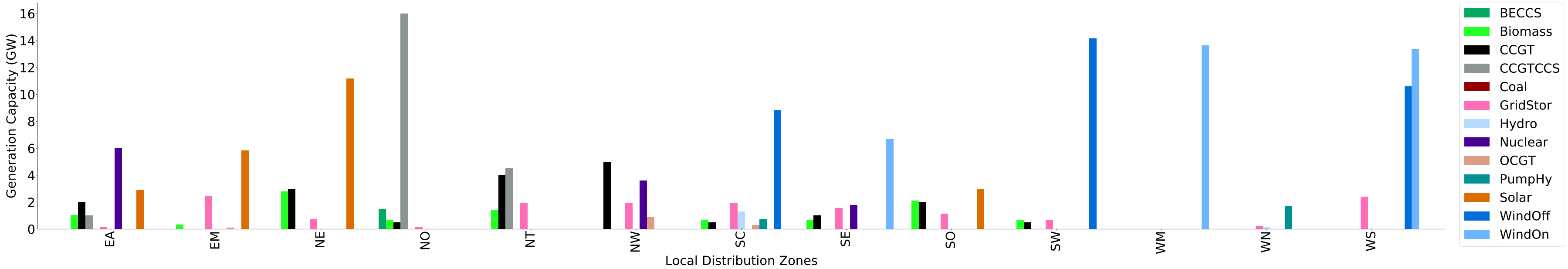

(d) Installed generation capacities across LDZs in 2053 (model output).

Figure 10: (continued) Optimised regional allocation of generation capacities for 2018-2053 for power sector only decarbonisation.

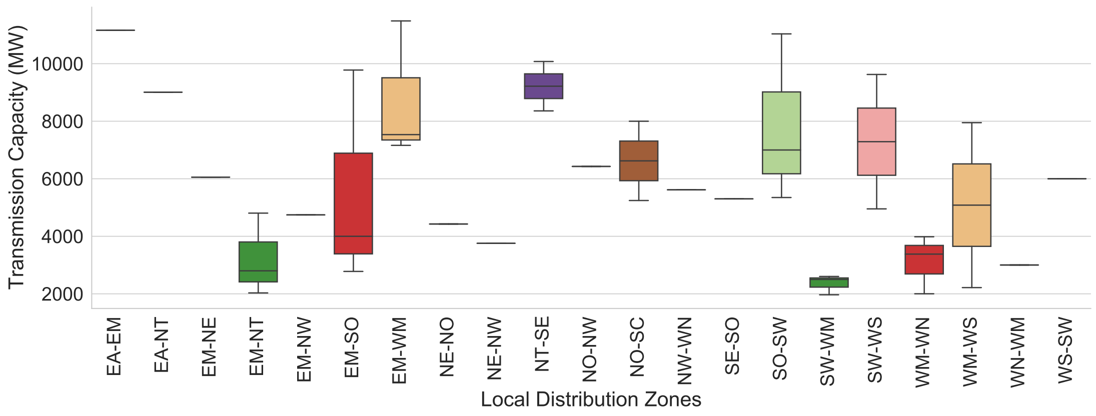

Figure 11: Boxplots of cross-regional transmission capacities in 2053 over different optimisation runs with different heat demand data from 2015-2018.

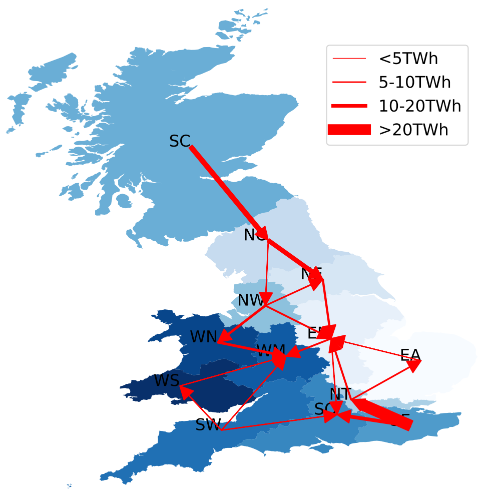

(a) Net transmission flows in 2028

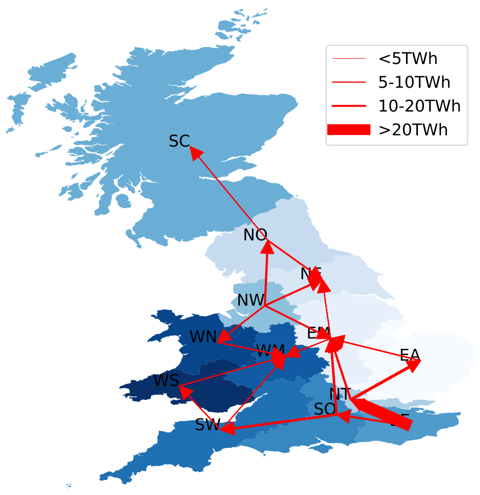

(b) Net transmission flows in 2038

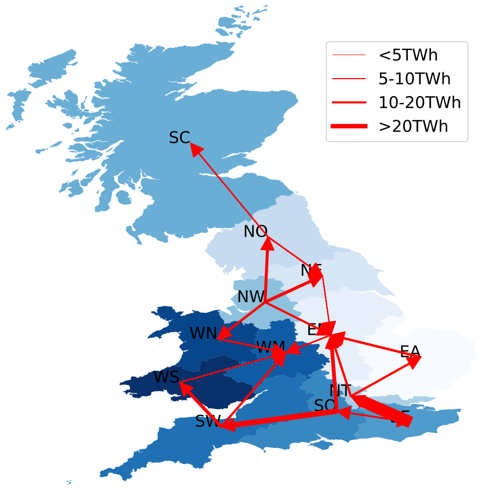

(c) Net transmission flows in 2053

Figure 12: Regional net transmission flows (TWh/year) with 2015 input data. Consistently the largest amount of transmission is observed from SE to NT. This is attributed to three factors: (i) NT absorbs significant amounts of the intermittent generation from SE (Solar & Onshore Wind) owing to the lack of within region installed capacity (cf. Fig. 9), (ii) increased demand of electricity due to heat electrification in NT and (iii) flexibility due to the presence of interconnection in SE. We also note that SC progressively towards mid-century becomes a net importer from NO mostly because of: (i) increased allocation of CCGT-CCS capacity in NO vs a predominantly intermittent capacity mix in SC & (ii) increased interconnection from SC to Norway.

Nomenclature

Sets

c Cluster of days

f Fuel

g, g' Geographic regions

h, h' Time-slices (hours)

j Technologies

j_e Electricity generation technologies

j_f Dynamic set of technologies j consuming fuel type f

j_h End-use heating technologies

j_s Energy storage technologies

j_{h^s} Thermal storage technologies

j_{rew} Renewable generation plants

j_{th} Thermal generation plants

t Time-steps (years)

Parameters

ϵ_f Fuel f emissions factor [tCO2e/MWh]

$\eta^{tech}_{g,j,c,h,t}$ Performance efficiency for end-use heating technologies [-]

$\overline{CO2^{elec}_t}$ CO2 emissions upper limit for the power sector in year t [tCO2e]

$\overline{CO2^{heat}_t}$ CO2 emissions upper limit for the heat sector in year t [tCO2e]

$Av_{g,j,c,h,t}$ Availability of renewable energy sources $j \in J_{rew}$ [%MW]

$BR_{j,t}$ Build rate of technology j at year t [-/MW]

$C^{CO_2}_t$ Carbon price at year t [£/tCO2e]

C^{CRTL} Unit cost of curtailed renewable power [£/MWh]

C^{fix}_j Fixed capital cost of technology j [£/MW]

C^{fix}_{tr} Fixed capital cost of transmission lines [£/MW]

$C^{fuel}_{f,t}$ Unit consumption cost of fuel f at year t [£/MWh]

$C^{INCX}_{i,g,c,h,t}$ Price of interconnected power from interconnector i to region g at time slice h and year t [£/MW]

C^{OM}_{j} Fixed O&M cost of technology j [£/MW]

$C^{OPRES_{down}}$ Value of loss load for shedding downward operating reserve requirements [£/MWh]

$C^{OPRES_{up}}$ Value of loss load for shedding upward operating reserve requirements [£/MWh]

C^{shut}_{j} Shut-down cost cost of technology j [£/MW]

C^{start}_{j} Start-up cost cost of technology j [£/MW]

$C^{STOR_{dn}}$ Unit cost of downward short term operating reserve [£/MW]

$C^{STOR_{up}}$ Unit cost of upward short term operating reserve [£/MW]

C^{var}_{j} Variable operating cost of technology j [£/MWh]

C^{VoLL} Unit cost of load shedding (Value of Loss Load) [£/MWh]

$CapUnit_{j}$ Unit capacity of thermal power plants [MW]

CCS^{rem}_{j} Capture rate of CO2 of technology j [%]

$COP_{g,c,h,t}$ Coefficient of performance for ASHPs [-]

$D^{error}_{c,h}$ Forecasting demand error for reserve calculations [%]

DF_{j} Derating factor of technology j for capacity reserve calculations [%]

DFC_{t} Discount factor [-]

DL Distribution power losses [%]

DT_{j} Minimum down-time of generation unit j [hours]

hcu_{j} Unit capacity of heat-end use technologies [MW]

$ICap_{i,g,t}$ Interconnection capacity between interconnector i, region g at year t [MW]

$InterLoss_{i,g}$ Interconnection transmission losses [%]

$LDD_{g,g'}$ Linear distance between the centroids of two geographic regions [km]

$nHg_{g,r}$ Number of domestic properties at region g and area r [-]

$P^{elec}_{g,c,h,t}$ Electricity demand [MWh]

P^{max}_{j} Maximum stable generation of generation plant [MWh]

P^{min}_{j} Minimum stable generation of generation plant [MWh]

PL_{j} Parasitic load factor % of gross generation [-]

r Discount rate [-]

RD_j Maximum ramp-down rate of generation unit j when committed [MWh]

$Res_{c,h}^{Error}$ Forecasting renewable generation error for reserve calculations [%]

RM Capacity reserve margin [%]

RU_j Maximum ramp-up rate of generation unit j when committed [MWh]

$SCap^{N-1}$ N-1 security capacity parametrisation for loss of larger power plant or interconnector [MW]

SD_j Maximum ramp-down rate of generation unit j when shutting-down [MWh]

ST_j^{out} Energy to power ratio for power storage [hours]

SU_j Maximum ramp-up rate of generation unit j when starting-up [MWh]

$T_{g,c,h,t}^{\alpha}$ Hourly ambient temperature [°C]

TES_j Energy to power ratio for thermal storage [hours]

$TL_{g,g'}$ Percentage of transmission losses per km of transmission [-]

tp_j Lifetime of technology j [years]

TRI^{up} Upper investment limit in new transmission capacity [MW]

UT_j Minimum up-time of generation unit j [hours]

WD_c Weight of each cluster (number of days) [-]

Integer variables

$CUnits_{g,j,r,t}^{heat}$ Number of new heat-end use units at region g within area r (on/off-gas grid) and year t

$CUnits_{g,j,t}$ Number of new thermal power generation units j in region g and year t

$DUnits_{g,j,r,t}^{heat}$ Number of decommissioned heat-end use units at region g within area r (on/off-gas grid) and year t

$DUnits_{g,j,t}$ Number of decommissioned thermal power generation units j in region g and year t

$NUnits_{g,j,r,t}^{heat}$ Number of installed heat-end use units in region g within area r (on/off-gas grid) and year t

$NUnits_{g,j,t}$ Number of installed thermal power generation units j in region g and year t

$u_{g,j,c,h,t}$ Number of thermal plants j$\in J_{th}$ in region g at time-slice h and year t committed

$v_{g,j,c,h,t}$ Number of thermal plants j$\in J_{th}$ in region g at time-slice h and year t starting-up

$w_{g,j,c,h,t}$ Number of thermal plants j$\in J_{th}$ in region g at time-slice h and year t shutting-down

Continuous variables

$Cap^{New}_{g,j,t}$ New investment capacity of non-thermal generation or storage technologies at region g and year t [MW]

$Cap_{g,j,t}$ Installed capacity of non-thermal generation or storage technologies at region g and year t [MW]

$CH_{g,j,c,h,t}$ Power charger to storage unit j at region g, time-slice h and year t [MWh]

${CO_2^{elec_{neg}}}_t$ Total negative CO2 emissions from the power sector at year t [tCO2e]

${CO_2^{elec}}_t$ Total CO2 emissions from the power sector at year t [tCO2e]

${CO_2^{heat}}_t$ Total CO2 emissions from the heat sector at year t [tCO2e]

$D_{g,c,h,t}$ Power demand (heat and non-heat) at region g, time-slice h and year t [MWh]

$DC_{g,j,c,h,t}$ Power discharged from storage unit j at region g, time-slice h and year t [MWh]

$HDem_{g,r,c,h,t}$ Hourly heat demand at region g and year t [MWh]

$l^{OPRES_{down}}_{c,h,t}$ Load shedding of downward reserve at region g, time-slice h and year t [MWh]

$l^{OPRES_{up}}_{c,h,t}$ Load shedding of upward reserve at region g, time-slice h and year t [MWh]

$LCurtail_{g,c,h,t}$ Curtailment of renewable energy resources connected to transmission at region g, time-slice h and year t [MWh]

$LShed_{g,c,h,t}$ Load shedding of electricity demand at region g, time-slice h and year t [MWh]

$OPRES^{down}_{g,c,h,t}$ Downward operating reserve requirement at region g, representative day c, time-slice h and year t [MWh]

$OPRES^{up}_{g,c,h,t}$ Upward operating reserve requirement at region g, representative day c, time-slice h and year t [MWh]

$P^{heat}_{g,r,c,h,t}$ Electricity load resulting from electrified heat [MWh]

$P_{g,j,c,h,t}$ Power generated at region g, by units j at time-slice h and year t [MWh]

$PeakD_t$ Peak power demand at year t [MWh]

$PIM_{i,g,c,h,t}$ Power imported at region g through interconnector at time-slice h and year t [MWh]

$Q^{dheat}_{g,r,h,t}$ Heat discharged from thermal storage at time-slice h at region g and year t [MWh]

$Q^{heat}_{g,r,j,c,h,t}$ Heat generated at time-slice h at region g and year t from technology j [MWh]

$Q^{sheat}_{g,r,h,t}$ Heat stored to thermal storage at time-slice h at region g and year t [MWh]

$r^{down}_{g,j,c,h,t}$ Downward reserve of thermal unit j at region g, time-slice h and year t [MWh]

$r^{up_{nonsync}}_{g,j,c,h,t}$ Upward nonsynchronous reserve of technology j at region g, time-slice h and year t [MWh]

$r^{up}_{g,j,c,h,t}$ Upward reserve of thermal unit j at region g, time-slice h and year t [MWh]

$S^{heat}_{g,r,c,h,t}$ Heat storage level at region g, grid area r, at time-slice h and year t [MWh]

$ST^{init}g, j, t$ Initial stored energy for technology j at region g and year t [MWh]

$TotCAPEX_t$ NPV of total electricity and heat system's capital costs at year t [£]

$TotOPEX_t$ NPV of total electricity and heat system's operating costs at year t [£]

$TR_{g,g',c,h,t}$ Power transmitted inter-regionally at time-slice h and year t [MWh]

$TRC_{g,g',t}$ Inter-regional transmission capacity [MW]

$TRI_{g,g',t}$ Inter-regional transmission capacity investement [MW]

TSC NPV of total electricity and heat system's costs [£]

$V^{elec}_{g,j,f,c,h,t}$ Fuel consumption of type f at region g by technology j, time-slice h and year t for power generation [MWh]

$V^{heat}_{g,j,f,c,h,t}$ Fuel consumption of type f at region g by technology j, time-slice h and year t for power generation [MWh]

References

1. Li, Z., Wu, W., Shahidehpour, M., Wang, J. & Zhang, B. Combined heat and power dispatch considering pipeline energy storage of district heating network. *IEEE Trans. Sustain. Energy* **7,** 12–22 (2016).
2. Ruhnau, O., Hirth, L. & Praktiknjo, A. Heating with wind: Economics of heat pumps and variable renewables. *Energy Economics* **92,** 104967 (2020).
3. Li, G. *et al.* Optimal dispatch strategy for integrated energy systems with CCHP and wind power. *Appl. Energy* **192,** 408–419 (2017).
4. Chen, X. *et al.* Increasing the flexibility of combined heat and power for wind power integration in China: Modeling and implications. *IEEE Trans. Power Syst.* **30,** 1848–1857 (2015).
5. Hedegaard, K. & Münster, M. Influence of individual heat pumps on wind power integration- Energy system investments and operation. *Energy Convers. Manag.* **75,** 673–684 (2013).
6. Hedegaard, K., Mathiesen, B. V., Lund, H. & Heiselberg, P. Wind power integration using individual heat pumps- Analysis of different heat storage options. *Energy* **47,** 284–293 (2012).

7. Munuera, L., Bradford, J., Kelly, N. & Hawkes, A. *The role of energy efficiency in decarbonising heat via electrification* ECEEE 2013 summer study. 2013.

8. Energy Saving Trust. *Getting Warmer: A Field Trial of Heat Pumps* Energy Saving Trust: London, UK. 2010.

9. Delta Energy & Environment. *Technical Feasibility of Electric Heating in Rural Off-Gas Grid Dwellings* BEIS Research Series, 24. 2018.

10. OFGEM. *The Decarbonisation of Heat* OFGEM's Future Insights Series. 2010.

11. Lowes, R., Rosenow, J., Qadrdan, M. & Wu, J. Hot stuff: Research and policy principles for heat decarbonisation through smart electrification. *Energy Research & Social Science* **70,** 101735 (2020).

12. McDowall, W. & Eames, M. Towards a sustainable hydrogen economy: A multi-criteria sustainability appraisal of competing hydrogen futures. *Int. J. Hydrogen Energy* **32,** 4611–4626 (2007).

13. CCC. *Hydrogen in a low-carbon economy.* Committee on Climate Change. 2018.

14. Burke, J. & Rooney, M. *Fuelling the future* Policy Exchange. 2018.

15. Dodds, P. E. & Demoullin, S. Conversion of the UK gas system to transport hydrogen. *Int. J. Hydrogen Energy* **38,** 7189–7200 (2013).

16. Dodds, P. E. & McDowall, W. The future of the UK gas network. *Energy Policy* **60,** 305–316 (2013).

17. Jalil-Vega, F. & Hawkes, A. Spatially resolved model for studying decarbonisation pathways for heat supply and infrastructure trade-offs. *Appl. Energy* **210,** 1051–1072 (2018).

18. Jalil-Vega, F. A. & Hawkes, A. D. Spatially Resolved Optimization for Studying the Role of Hydrogen for Heat Decarbonization Pathways. *ACS Sustain. Chem. Eng.* **6,** 5835–5842 (2018).

19. Quiggin, D. & Buswell, R. The implications of heat electrification on national electrical supply-demand balance under published 2050 energy scenarios. *Energy* **98,** 253–270 (2016).

20. Heinen, S., Turner, W., Cradden, L., McDermott, F. & O'Malley, M. Electrification of residential space heating considering coincidental weather events and building thermal inertia: A system-wide planning analysis. *Energy* **127,** 136–154 (2017).

21. Eyre, N. & Baruah, P. Uncertainties in future energy demand in UK residential heating. *Energy Policy* **87,** 641–653 (2015).

22. Heinen, S., Burke, D. & O'Malley, M. Electricity, gas, heat integration via residential hybrid heating technologies- An investment model assessment. *Energy* **109,** 906–919 (2016).

23. Barton, J. *et al.* The evolution of electricity demand and the role for demand side participation, in buildings and transport. *Energy Policy* **52,** 85–102 (2013).

24. Lund, H. & Mathiesen, B. V. Energy system analysis of 100% renewable energy systems—The case of Denmark in years 2030 and 2050. *Energy* **34,** 524–531 (2009).

25. Heaton, C. Modelling low-carbon energy system designs with the ETI ESME model. *Energy Technologies Institute,* 1–28 (2014).

26. Zhang, X., Strbac, G., Teng, F. & Djapic, P. Economic assessment of alternative heat decarbonisation strategies through coordinated operation with electricity system–UK case study. *Appl. Energy* **222,** 79–91 (2018).

27. Sansom, R. & Strbac, G. *The impact of future heat demand pathways on the economics of low carbon heating systems* in *BIEE-9th Academic conference* (2012), 1–10.

28. Dodds, P. E. Integrating housing stock and energy system models as a strategy to improve heat decarbonisation assessments. *Appl. energy* **132,** 358–369 (2014).

29. Chaudry, M., Jenkins, N., Qadrdan, M. & Wu, J. Combined gas and electricity network expansion planning. *Applied Energy* **113,** 1171–1187 (2014).

30. McKenna, R. C. & Norman, J. B. Spatial modelling of industrial heat loads and recovery potentials in the UK. *Energy Policy* **38,** 5878–5891 (2010).

31. Rattner, A. S. & Garimella, S. Energy harvesting, reuse and upgrade to reduce primary energy usage in the USA. *Energy* **36,** 6172–6183 (2011).

32. Bloess, A., Schill, W.-P. & Zerrahn, A. Power-to-heat for renewable energy integration: A review of technologies, modeling approaches, and flexibility potentials. *Appl. Energy* **212,** 1611–1626 (2018).

33. Qadrdan, M., Fazeli, R., Jenkins, N., Strbac, G. & Sansom, R. Gas and electricity supply implications of decarbonising heat sector in GB. *Energy* (2018).

34. Love, J. *et al.* The addition of heat pump electricity load profiles to GB electricity demand: Evidence from a heat pump field trial. *Appl. Energy* **204,** 332–342 (2017).

35. Element Energy & E4tech. *Cost analysis of future heat infrastructure options* Report for National Infrastructure Commission. 2018.

36. Imperial College London. *Analysis of Alternative UK Heat Decarbonisation Pathways.* Report for Committee on Climate Change. 2018.

37. KPMG. 2050 Energy Scenarios. *Energy Networks Association,* 1–72 (2016).

38. Valor, E., Meneu, V. & Caselles, V. Daily air temperature and electricity load in Spain. *Journal of applied Meteorology* **40,** 1413–1421 (2001).

39. Hirth, L., Mühlenpfordt, J. & Bulkeley, M. The ENTSO-E Transparency Platform–A review of Europe's most ambitious electricity data platform. *Applied energy* **225,** 1054–1067 (2018).

40. National Grid. *Gas demand forecasting methodology* National Grid Gas. 2016.

41. OFGEM. *Analysis of the first phase of the Electricity Balancing Significant Code Review* OFGEM. 2018.

42. National Grid Electricity plc. *Special Condition 2K.4 – Transmission Losses Report, Reporting Period 1 April 2019 to 31 March 2020* National Grid Electricity plc. 2018.

43. Heuberger, C. F., Bains, P. K. & Mac Dowell, N. The EV-olution of the power system: A spatio-temporal optimisation model to investigate the impact of electric vehicle deployment. *Applied Energy* **257,** 113715 (2020).

44. Staffell, I., Brett, D., Brandon, N. & Hawkes, A. A review of domestic heat pumps. *Energy Environ Sci.* **5,** 9291–9306 (2012).

45. Vorushylo, I., Keatley, P., Shah, N., Green, R. & Hewitt, N. How heat pumps and thermal energy storage can be used to manage wind power: A study of Ireland. *Energy* **157,** 539–549 (2018).

46. Bach, B. *et al.* Integration of large-scale heat pumps in the district heating systems of Greater Copenhagen. *Energy* **107,** 321–334 (2016).

47. Delta Energy & Environment Ltd. *Evidence Gathering: Thermal Energy Storage (TES) Technologies* tech. rep. (Department of Business, Energy & Industrial Strategy, 2018).

48. Baldwin, C., Dale, K. & Dittrich, R. A study of the economic shutdown of generating units in daily dispatch. *Trans. Amer. Inst. Elec. Eng. :Power App. Syst.* **78,** 1272–1282 (1959).

49. Sheble, G. B. & Fahd, G. N. Unit commitment literature synopsis. *IEEE Trans. Power Syst.* **9,** 128–135 (1994).

50. Saravanan, B., Das, S., Sikri, S. & Kothari, D. A solution to the unit commitment problem—a review. *Front. Energy* **7,** 223–236 (2013).

51. Lee, J., Leung, J. & Margot, F. Min-up/min-down polytopes. *Discrete Optim.* **1,** 77–85 (2004).

52. Rajan, D. & Takriti, S. Minimum up/down polytopes of the unit commitment problem with start-up costs. *IBM Res. Rep,* 1–14 (2005).

53. National Grid. *Electricity Capacity Report* National Grid ESO. 2019.

54. CCC. *The Sixth Carbon Budget, The UK's Path to Net Zero.* Committee on Climate Change. `https://www.theccc.org.uk/publication/sixth-carbon-budget/`. 2020.

55. National Grid. *Data Portal: Historic Demand Data.* National Grid ESO. `https://data.nationalgrideso.com/demand/historic-demand-data`. 2020.

56. BEIS. *Regional and local authority electricity consumption statistics.* Department of Business, Energy & Industrial Strategy. `https://www.gov.uk/government/statistical-data-sets/regional-and-local-authority-electricity-consumption-statistics`. 2019.

57. National Grid. *Electricity Ten Year Statement* National Grid ESO. 2020.

58. Zeyringer, M., Price, J., Fais, B., Li, P.-H. & Sharp, E. Designing low-carbon power systems for Great Britain in 2050 that are robust to the spatiotemporal and inter-annual variability of weather. *Nature Energy* **3,** 395–403 (2018).

59. Egerer, J. *Open source electricity model for Germany (ELMOD-DE)* tech. rep. (DIW Data Documentation, 2016).

60. BEIS. *Digest of UK Energy Statistics (DUKES): electricity. Power stations in the United Kingdom.* Department of Business, Energy & Industrial Strategy. https://www.gov.uk/government/statistics/electricity-chapter-5-digest-of-united-kingdom-energy-statistics-dukes. 2019.

61. Bews, P. *Electricity Generation Costs and Hurdle Rates: A report prepared for the Department of Energy and Climate Change* tech. rep. (LeighFisher Ltd, 2016).

62. MacDonald, A. E. *et al.* Future cost-competitive electricity systems and their impact on US CO_2 emissions. *Nature Climate Change* **6,** 526–531 (2016).

63. Brinckerhoff, P. Electricity generation cost model- 2013 Update Of Non-Renewable Technologies. *Department of Energy and Climate Change* (2013).

64. Schill, W.-P., Pahle, M. & Gambardella, C. Start-up costs of thermal power plants in markets with increasing shares of variable renewable generation. *Nature Energy* **2,** 1–6 (2017).

65. Schröder, A., Kunz, F., Meiss, J., Mendelevitch, R. & Von Hirschhausen, C. *Current and prospective costs of electricity generation until 2050* tech. rep. (DIW data documentation, 2013).

66. Fajardy, M. & Mac Dowell, N. Can BECCS deliver sustainable and resource efficient negative emissions? *Energy & Environmental Science* **10,** 1389–1426 (2017).

67. BEIS. *Electricity Generation Costs.* Department of Business, Energy & Industrial Strategy. https://www.gov.uk/government/publications/beis-electricity-generation-costs-2020. 2020.

68. Zakeri, B. & Syri, S. Electrical energy storage systems: A comparative life cycle cost analysis. *Renewable and sustainable energy reviews* **42,** 569–596 (2015).

69. Poyry, C. *Benefits of GB Interconnection, 2016*

70. Redpoint. *Impacts of further electricity interconnection on Great Britain: A Report by Redpoint Energy Limited, a business of Baringa Partners,for the Department of Energy and Climate Change (DECC).* Department of Energy and Climate Change. 2013.
